# The penultimate step of proteasomal ATPase assembly is mediated by a switch dependent on the chaperone Nas2

**DOI:** 10.1016/j.jbc.2023.102870

**Published:** 2023-01-05

**Authors:** Suganya Sekaran, Soyeon Park

**Affiliations:** Department of Molecular Cellular and Developmental Biology, University of Colorado Boulder, Boulder, Colorado, USA

**Keywords:** proteasome, chaperone, molecular chaperone, ATPase associated with diverse cellular activities, protein assembly, Nas2, CP, core particle, pu-base, penultimate base, RP, regulatory particle, TBST, Tris-buffered saline with 0.1% Tween-20, YPD, yeast extract–peptone–dextrose

## Abstract

The proteasome holoenzyme is a complex molecular machine that degrades most proteins. In the proteasome holoenzyme, six distinct ATPase subunits (Rpt1 through Rpt6) enable protein degradation by injecting protein substrates into it. Individual Rpt subunits assemble into a heterohexameric “Rpt ring” in a stepwise manner, by binding to their cognate chaperones. Completion of the heterohexameric Rpt ring correlates with release of a specific chaperone, Nas2; however, it is unclear whether and how this event may ensure proper Rpt ring assembly. Here, we examined the action of Nas2 by capturing the poorly characterized penultimate step of heterohexameric Rpt ring assembly. For this, we used a heterologous *Escherichia coli* system coexpressing all Rpt subunits and assembly chaperones as well as *Saccharomyces cerevisiae* to track Nas2 actions during endogenous Rpt ring assembly. We show that Nas2 uses steric hindrance to block premature progression of the penultimate step into the final step of Rpt ring assembly. Importantly, Nas2 can activate an assembly checkpoint *via* its steric activity, when the last ATPase subunit, Rpt1, cannot be added in a timely manner. This checkpoint can be relieved *via* Nas2 release, when Nas2 recognizes proper addition of Rpt1 to one side of its cognate Rpt5, and ATP hydrolysis by Rpt4 on the other side of Rpt5, allowing completion of Rpt ring assembly. Our findings reveal dual criteria for Nas2 release, as a mechanism to ensure both the composition and functional competence of a newly assembled proteasomal ATPase, to generate the proteasome holoenzyme.

The proteasome holoenzyme is responsible for degrading most proteins in the cell. It forms *via* association of three subcomplexes: the 9-subunit base, 9-subunit lid, and 28-subunit core particle (CP) (1, 2). In the proteasome holoenzyme, the base subcomplex recognizes polyubiquitinated proteins, unfolds, and translocates them into the barrel-shaped CP, where their degradation occurs (3, 4). During this process, the lid cleaves the polyubiquitin chain from the protein substrate, facilitating substrate translocation into the CP. These multistep events of protein degradation occur through a series of specific conformational changes between the base and lid and also relative to the CP (5, 6, 7, 8, 9). The base coordinates these conformational changes using ATP hydrolysis through its six distinct ATPase subunits (Rpt1 through Rpt6), which are arranged into a ring-shaped complex, forming a key structure of the base (6, 7, 8, 10). The heterohexameric ATPase “ring” provides the lateral binding site for the lid, generating a base–lid complex referred to as the regulatory particle (RP) (10, 11). In the RP, the heterohexameric ATPase ring docks into the cylindrical end of the CP, mediating RP–CP association to complete proteasome holoenzyme assembly (6, 7, 8, 10). RPs can associate with either one or both ends of the CP, generating a singly or doubly capped proteasome holoenzyme termed RP_1_–CP or RP_2_–CP (1, 2). These features of the base emphasize its importance for both assembly and function of the proteasome holoenzyme.

Base assembly relies on four dedicated chaperone proteins, Nas2, Hsm3, Rpn14, and Nas6, which are conserved between yeast and humans (12, 13, 14, 15, 16, 17, 18). Each specific chaperone acts by binding to its cognate Rpt protein: Nas2–Rpt5, Hsm3–Rpt1, Rpn14–Rpt6, and Nas6–Rpt3 (12, 13, 14, 16, 17). Three distinct modules then form, with each module containing one or two chaperones: Nas2–Rpt5–Rpt4, Hsm3–Rpt1–Rpt2–Rpn1, and Rpn14–Rpt6–Rpt3–Nas6. Chaperones together facilitate the three modules to associate into a heterohexameric Rpt ring, in which individual Rpt subunits are specifically arranged as Rpt5–Rpt4–Rpt3–Rpt6–Rpt2–Rpt1. During this process, the CP can transiently serve as a template to nucleate a heterohexameric Rpt ring (15, 16) and also to help facilitate conformational changes of the base complex as it proceeds into the proteasome holoenzyme (19, 20). In the current model, binding of each specific chaperone to their cognate Rpt protein sterically hinders the premature or inappropriate association with the CP, until base assembly is complete, thereby preventing formation of defective proteasome complexes (15, 16, 19, 20, 21, 22, 23). Upon completion of the proteasome holoenzyme, the chaperones are evicted (15, 16, 21, 24).

Although the base complex en route to the proteasome holoenzyme contains three chaperones (Rpn14, Nas6, and Hsm3), it excludes a fourth chaperone, Nas2 (25, 26, 27). Nas2 is proposed to release, because of its steric conflict with the fully formed base, implying that steric effect of Nas2 might facilitate proper assembly of the base (25). However, it remains unclear whether and how Nas2 regulates the actual process of base assembly. Contrary to multiple approaches for examining the base-binding chaperones (Rpn14, Nas6, and Hsm3) during the progression of the assembled base into the proteasome holoenzyme (12, 16, 17, 20, 21, 22, 24, 28), approaches for examining Nas2 have been challenging to establish, because of the rapid progression and low level of the assembly intermediates, prior to completion of the base (15, 17). For this reason, one of the least characterized segments of base assembly remains the penultimate step involving Nas2. At this step, the Nas2–Rpt5–Rpt4 module is proposed to associate with the Rpn14–Rpt6–Rpt3–Nas6 module, which is considered as a “seed” of base assembly but is not readily detectable (17, 26, 27). Nas2 is then thought to release, upon incorporation of the Hsm3–Rpn1–Rpt1–Rpt2 module, completing the base (25). The Nas2 ortholog in humans, p27, is also proposed to release upon completion of base assembly, with the p27–Rpt5–Rpt4 module being added last to complete the base (13, 18). Although some potential difference might exist in the order of base assembly between yeast and humans, the human ortholog of Nas2 also seems to specifically contribute to proper completion of the base itself. Our studies here examine this poorly characterized segment of base assembly to investigate whether and how Nas2 may ensure proper completion of base assembly.

We examined Nas2 actions by capturing the penultimate step of base assembly using both a heterologous *Escherichia coli* system and budding yeast, *Saccharomyces cerevisiae*. We find that Nas2 sterically hinders addition of other subunits until the penultimate base (pu-base) forms. Nas2 allows for completion of the base complex, specifically when ATP hydrolysis by Rpt4 occurs, providing a signal for Nas2 release and proper incorporation of Rpt1. Through this mechanism, Nas2 may ensure both subunit composition and functional competence of the newly assembled base for generating a proteasome holoenzyme.

## Results

### Isolation of the pu-base complex using a heterologous *E. coli* system

To investigate early stage base assembly events that are not readily detected in yeast, we used a heterologous system in *E. coli* coexpressing nine base subunits and four assembly chaperones (29). In this system, two different affinity tags, one FLAG and six histidines (His_6_), were appended to Rpt1 and Rpt3, respectively. A functional base can be isolated *via* two consecutive affinity purifications, His_6_-Rpt3 followed by FLAG-Rpt1 as a bait, since these two Rpt proteins form two different modules first and later join to complete the base complex (1, 2, 29). Here, we took advantage of this system and conducted two parallel His_6_-Rpt3 *versus* FLAG-Rpt1 affinity purifications, to obtain assembly intermediates that are en route to the base but are difficult to isolate because of their low level or rapid progression during endogenous base assembly.

**Figure 1:**
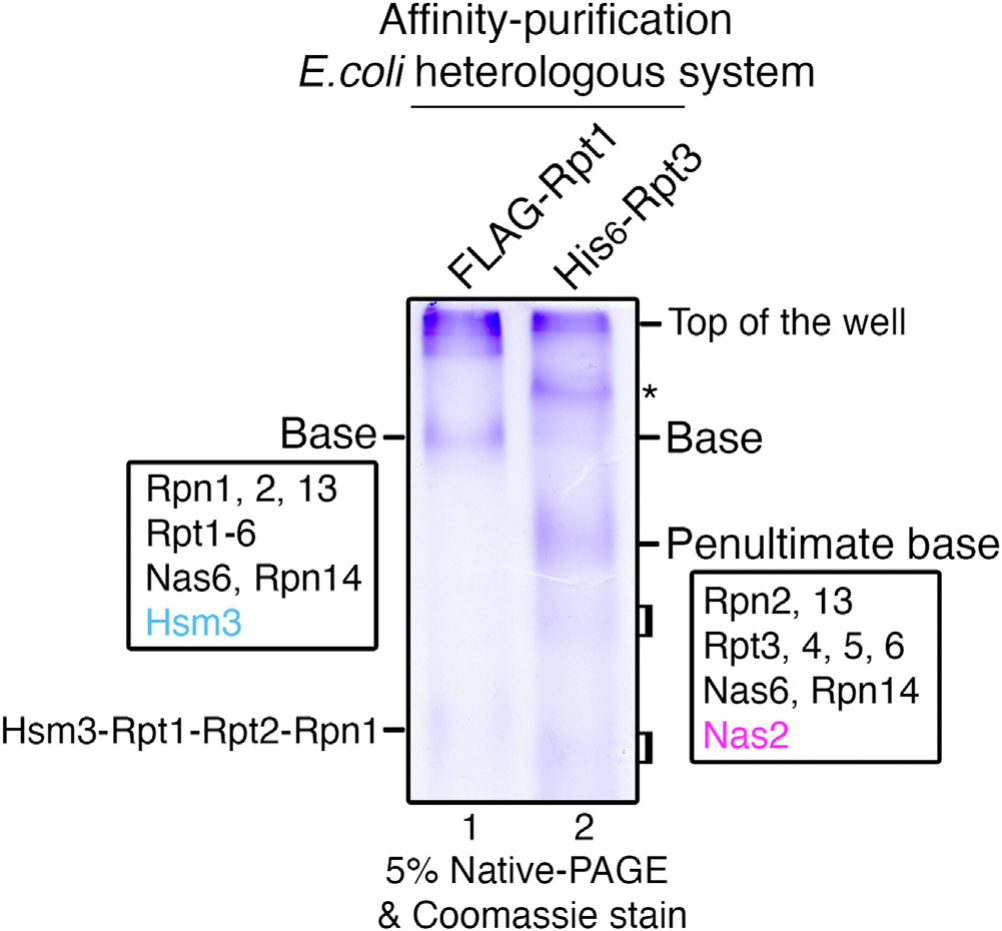
**Isolation of the penultimate base (pu-base) complex using the heterologous *Escherichia coli* system.** The pu-base can be affinity purified using His_6_-tagged Rpt3 as a bait in the heterologous *E. coli* system, together with the base, which can be isolated using FLAG-tagged Rpt1 (29). Purified proteins (10 μg) were subjected to 5% native-PAGE, followed by Coomassie blue staining. Subunit composition of the base and pu-base was identified by excising the corresponding bands for a proteomics analysis (Table S1). Two broad and faint bands below the pu-base (*brackets*, lane 2) are not reactive with Rpt6, suggesting that they are not likely to be the Rpn14–Rpt6–Rpt3–Nas6 module (Fig. S1, lane 2). *Asterisk*, nonspecific binding of *E. coli* proteins.

Native gel analysis of FLAG-Rpt1 affinity-purified materials exhibited one major complex. This complex was identified as the fully formed base, as confirmed by proteomics analysis (Fig. 1, lane 1 and Table S1). All nine integral base subunits were identified, together with three assembly chaperones (Nas6, Rpn14, and Hsm3). The fourth chaperone, Nas2, was absent in the base, consistent with the evidence suggesting that Nas2 releases prior to completion of the base (Fig. 1) (25, 26, 27). In addition to the fully formed base complex, a trace amount of the other Rpt1-containing complex, the Hsm3–Rpt1–Rpt2–Rpn1 module, was detected by immunoblotting (Fig. 1, lane 1 and Fig. S1, lane 1). These results are consistent with the established order of base assembly steps, where Rpt1 exists mainly in two complexes: the Hsm3–Rpt1–Rpt2–Rpn1 module and the fully formed base (12, 14, 17).

On the other hand, our native gel analysis of His_6_-Rpt3 affinity-purified material exhibited several bands (Fig. 1, lane 2). The slowest-migrating band was identified as nonspecific *E. coli* proteins (Fig. 1, *asterisk*, lane 2), and the faint band underneath it was the base complex, as seen in FLAG-Rpt1 affinity purification (Fig. S1, lane 2). Importantly, a more prominent band below the base was identified as a 9-subunit complex, consisting of two non-ATPase subunits (Rpn2 and Rpn13), four ATPase subunits (Rpt3, Rpt4, Rpt5, and Rpt6), and three chaperones (Nas6, Rpn14, and Nas2) (Fig. 1, lane 2; Table S1; Fig. S1, lane 2). Based on the established order of base assembly in yeast (1, 2), this complex contains all base components, except the last module, Hsm3–Rpt1–Rpt2–Rpn1, whose incorporation completes base assembly. Thus, we will refer to this complex as the pu-base henceforth.

The penultimate step of base assembly has been poorly characterized, because of its low steady-state level during endogenous proteasome assembly (17). Now that we can capture the pu-base complex (Fig. 1, lane 2), we examined how this step might contribute to proper completion of base assembly. In a previous study using a structural model, Nas2 was proposed to exhibit steric conflict with the fully formed base, as its binding to the cognate Rpt5 subunit masks the Rpt1-interacting surface (25). However, an important and unanswered question remains, as to whether and how such steric effect of Nas2 might regulate the actual process of base assembly. We sought to test Nas2 actions using the heterologous system, since the penultimate and fully formed base could be isolated in parallel, through His_6_-Rpt3 and FLAG-Rpt1 affinity purification, respectively (Fig. 1).

### Nas2 blocks the premature progression of the pu-base into the base

Based on the proposed steric conflict between Nas2 and the fully formed base (25), we tested whether Nas2 could restrict the progression of base assembly. If so, the absence of Nas2 should increase the progression of base assembly. This aspect of Nas2 action could not be directly tested during endogenous base assembly, since the base continuously proceeds to the proteasome holoenzyme (lid–base–CP complex) by associating with the lid and CP. Since both lid and CP are absent in *E. coli*, the base does not progress into higher-order complexes and instead accumulates as a final complex, allowing us to test this prediction.

**Figure 2:**
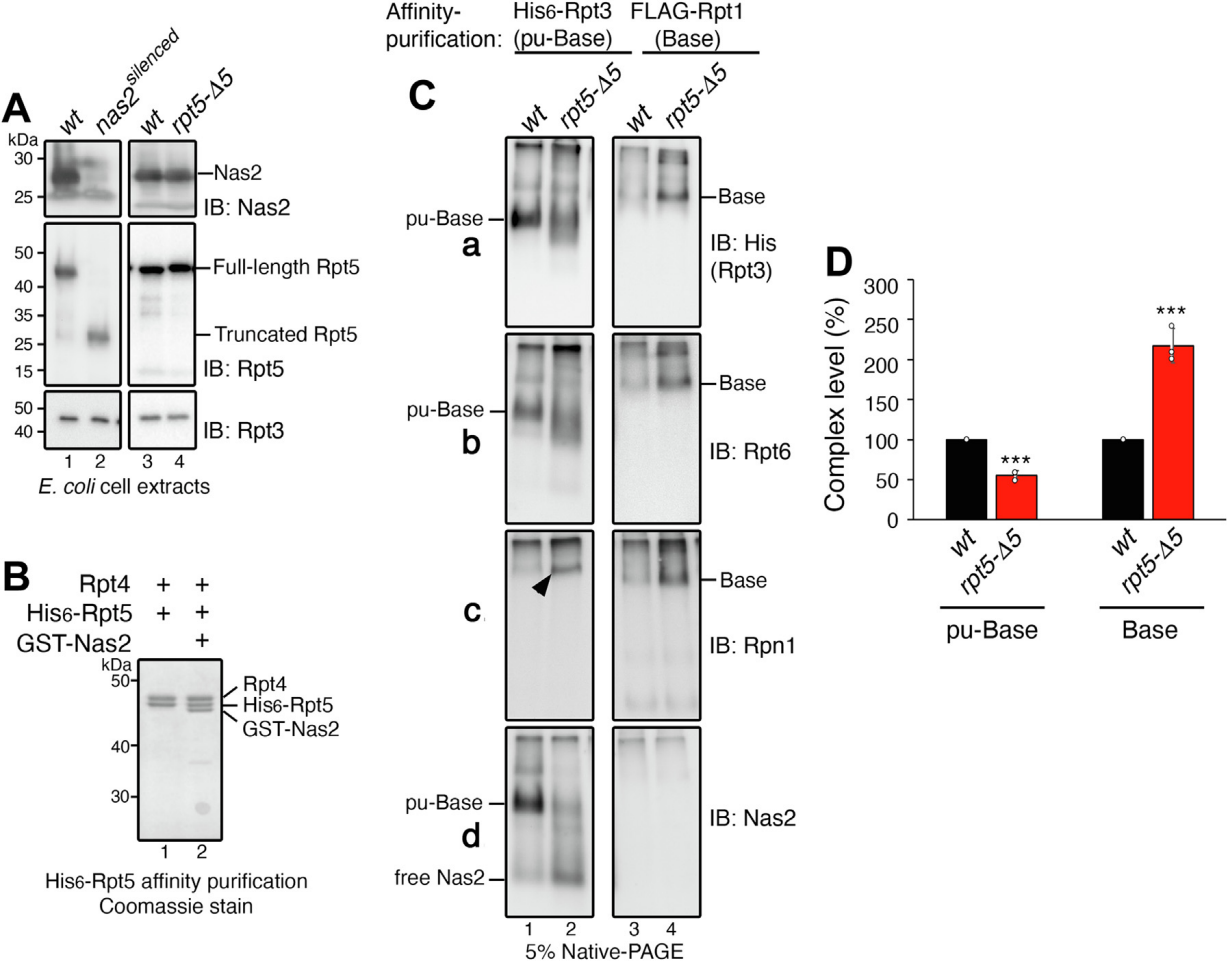
**Nas2 blocks the premature progression of the penultimate base (pu-base) into the base.***A*, validation that both Nas2 and Rpt5 are expressed normally in the *rpt5-Δ5* mutant, which specifically disrupts Nas2 binding to Rpt5 (31). Nas2 silencing (*nas2*^*silenced*^) leads to an artifact, Rpt5 truncation, in the *Escherichia coli* heterologous system coexpressing all nine subunits of the base and four assembly chaperones. Whole-cell extracts (15 μg) from the *E. coli* cells were analyzed by 10% Bis–Tris SDS-PAGE followed by immunoblotting for the indicated proteins. Rpt3, a subunit of the base and a loading control. *B*, Rpt5 stably forms the Nas2–Rpt5–Rpt4 module in *E. coli* cells coexpressing two Rpt subunits, Rpt4 and His_6_-tagged Rpt5, with or without Nas2. Affinity-purified protein (5 μg) using His_6_-tagged Rpt5 as a bait was subjected to 10% Bis–Tris SDS-PAGE and Coomassie stain. *C*, the pu-Base more readily progresses into the base, upon disruption of Nas2 binding to the pu-Base, as in the *rpt5-Δ5* mutant. Affinity purification was conducted in parallel, using His_6_-tagged Rpt3 and FLAG-tagged Rpt1, to obtain the pu-Base and the base, respectively. Affinity-purified proteins (4 μg) were subjected to 5% native-PAGE and immunoblotting for indicated subunits of the base (Rpt3, Rpt6, and Rpn1) and Nas2. *D*, quantification of the relative levels of the pu-Base and the base, using the data as in *C*, panels [a] and [b] (mean ± SD, n = 3 biological replicates, ∗∗∗*p* < 0.0005).

To abolish the proposed Nas2 steric hindrance, we silenced Nas2 expression in the heterologous system by introducing a premature stop codon into Nas2 (Fig. 2*A*, lane 2, *nas2*^*silenced*^). However, silencing Nas2 led to a defect in synthesis or stability of its cognate Rpt5, as seen from the truncated Rpt5 protein (Fig. 2*A*, lane 2). Such a defect on Rpt5 protein was specific to the *E. coli* heterologous system, likely because of a large demand of coexpressing all 9-base subunits. It should be noted that this effect was not seen during proteasome assembly in yeast, since endogenous Rpt5 was expressed normally and remained stable without Nas2 (30). As an alternative approach to abolish Nas2 steric hindrance, we disrupted only the binding of Nas2 to Rpt5, by deleting a portion of Nas2 binding site on the Rpt5 C-terminal tail (the last five amino acids, indicated as *rpt5-Δ5*) (29, 31). We confirmed that both Rpt5 and Nas2 were expressed normally in the *rpt5-Δ5* mutants, as in the wildtype cells (Fig. 2*A*, lanes 3 and 4). These data are consistent with the current model that the base assembly chaperones organize base assembly events, rather than regulating subunit stability or synthesis (1, 2). In fact, when only Rpt5 and Rpt4 was expressed in *E. coli*, instead of all nine subunits of the base, Rpt5 stably existed irrespective of Nas2, forming the Rpt5–Rpt4 module (Fig. 2*B*, compare lane 2 to 1).

To examine whether Nas2 can regulate base assembly *via* its steric effect, we isolated the pu-base *via* His_6_-Rpt3 affinity purification from the heterologous system. We confirmed that Nas2 binding to the pu-base was substantially reduced in the *rpt5-Δ5* mutant, as compared with its wildtype counterpart (Fig. 2*C*, [d], compare lane 2 to 1). Some free Nas2 was detected toward the bottom of the native gel, likely because of dissociation of residual Nas2 from the pu-base (Fig. 2*C*, [d], lane 2). As compared with wildtype, the yield of the pu-base in the *rpt5-Δ5* mutant was decreased to 50% of wildtype (Fig. 2*C*, [a, b], compare lane 1 to 2; see quantification in Fig. 2*D*). Some smeary appearance of the pu-base complex might indicate more heterogenous conformations because of loss of Nas2 binding in this complex, since protein mobility on a native gel is influenced by not only its molecular mass but also conformation (32, 33).

Notably, a decrease in the yield of the pu-base in the *rpt5-Δ5* mutants was accompanied by a corresponding increase in the yield of the base complex, as seen from immunoblotting for representative base subunits, Rpt3, Rpt6, and Rpn1 (Fig. 2*C*, lane 4 in [a, b, c]; see quantification in Fig. 2*D*). A twofold increase in the level of the base fits with the observed twofold decrease in the level of the pu-base in the *rpt5-Δ5* mutants (Fig. 2*D*). This result supports that the progression of the pu-base into the base was accelerated without Nas2. Also, the penultimate step of base assembly in the *rpt5-Δ5* mutants already incorporated Rpn1, exhibiting Rpn1 signal in a slowly migrating complex (Fig. 2*C*, [c], *arrowhead* in lane 2). Given that Rpn1 was normally absent in the pu-base (Fig. 1, lane 2), this result suggests that Rpn1 prematurely incorporated at the penultimate step of base assembly in the *rpt5-Δ5* mutants. Thus, without Nas2, the pu-base can proceed to the base more readily. These data provide experimental evidence that Nas2 sterically restricts the progression of the penultimate step to the final step for proper completion of the base, likely acting as a potential checkpoint.

### The pu-base forms during endogenous base assembly in an Nas2-dependent manner

**Figure 3:**
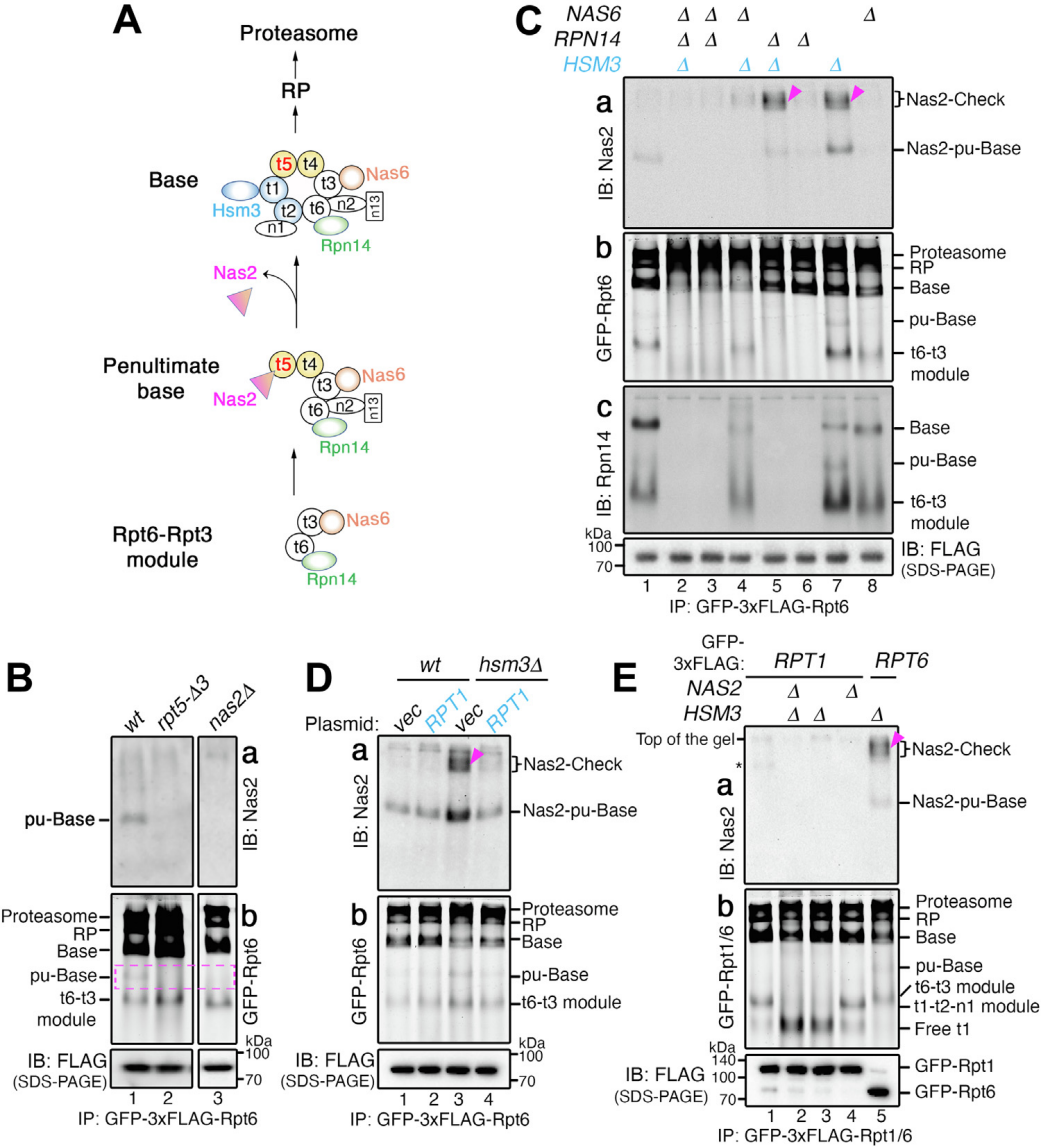
**Nas2 activates a checkpoint during endogenous base assembly, when Rpt1 cannot be properly incorporated.***A*, *cartoon* showing the sequence of chaperone-mediated base assembly. Six distinct Rpt subunits (indicated as t1 through t6) undergo stepwise assembly into the base complex. The Rpt6–Rpt3 module with two chaperones, Rpn14 and Nas6, forms first and then associates with the Rpt5–Rpt4 module harboring the Nas2 chaperone. Two non-ATPase subunits, Rpn2 and Rpn13 (n2 and n13), are then added (1, 2). This penultimate base (pu-base) complex may utilize steric effects of Nas2 against Rpt1 (25) and of Nas6 against the CP (16, 19, 20). The base complex completes upon incorporation of the Rpt1–Rpt2–Rpn1 module *via* Hsm3 and release of Nas2 (25, 26). For clarity, this *cartoon* focuses on chaperone actions during early stage base assembly and does not show chaperone actions during the progression of the base into a proteasome holoenzyme. *B*, the endogenous pu-base forms in an Nas2-dependent manner. To capture the pu-base and the preceding Rpt6–Rpt3 module in *A*, affinity purification was conducted using Rpt6 harboring a GFP-3xFLAG tag. Affinity-purified proteins (7.5 μg) were subjected to 5% native-PAGE, followed by immunoblotting for Nas2 [a] and Typhoon imaging for detection of GFP fluorescence on Rpt6 [b]. Anti-FLAG immunoblot was used as a loading control for FLAG pulldown in *B*–*E*. *C*, Nas2 activates an assembly checkpoint, upon disruption of Hsm3 activity during endogenous base assembly. Nas2–Check complexes (*arrowheads*) are detected *via* affinity purification using Rpt6 with GFP-3xFLAG tag. Affinity-purified proteins (7.5 μg) were subjected to 5% native-PAGE, followed by immunoblotting for Nas2 and Rpn14 [a and c] and by detection of GFP fluorescence to visualize Rpt6-containing complexes [b]. *D*, Nas2–Checkpoint can be neutralized by compensating for disruption of Hsm3 activity, through Rpt1 overexpression. Empty vector (vec) or low-copy expression plasmid harboring *RPT1* was introduced into wildtype and *hsm3Δ* cells. Experiments were conducted as in *B*. *E*, Rpt1 cannot incorporate into the Nas2–Check complex because of steric hindrance of Nas2 against Rpt1. Experiments were conducted as in *B*, for the pulldowns with GFP-3xFLAG-tagged Rpt1 (lanes 1–4) and GFP-3xFLAG-tagged Rpt6 (lane 5) using native-PAGE followed by immunoblotting [a] and GFP fluorescence detection [b]. Lane 5 provides a positive control for the Nas2–Check complex (*arrowhead*) showing incorporation of Rpt6 into this complex. *Asterisk* in [a] is a nonspecific signal (Fig. S4). CP, core particle.

Based on our data supporting Nas2 action as a checkpoint for base assembly in the heterologous system, we sought to investigate whether and how Nas2 may provide a checkpoint during endogenous base assembly in yeast, *S. cerevisiae*. We first tested whether the endogenous pu-base could be captured, since it has not been readily detectable as a discrete complex on a native gel. For this, we used the established strain harboring GFP-3xFLAG tagged Rpt6 (17, 34). Since this strategy has been used to track Rpt6 incorporation into late-stage assembly intermediates, such as the base and RP (17, 34), it should be able to also track the early segment of base assembly—the pu-base and the preceding Rpt6-Rpt3 module (Fig. 3*A*). Following affinity purification using the 3xFLAG tag on Rpt6, GFP fluorescence readily visualized Rpt6 incorporation into more abundant late-stage assembly intermediates, base and RP, and then into proteasome holoenzymes, migrating closely together toward the top of the gel (Fig. 3*B*, [b]); this segment of base assembly into the proteasome holoenzyme has been well established (17, 34).

Importantly, the endogenous pu-base was detected, albeit at a low level, migrating below the base in wildtype cells (Fig. 3*B*, see pu-Base, lane 1 in [a, b]); its subunit composition was validated by a proteomics analysis (Table S2). It is discernible that the existence of the pu-base was dependent on the Nas2 chaperone, since this complex was not detectable upon disruption of Nas2 binding to the Rpt5 C terminus (*rpt5-Δ3*) or complete deletion of *NAS2* (*nas2Δ*) (Fig. 3*B*, lanes 2 and 3 in [a, b]; *rpt5-Δ3* lacks the last three amino acids as in Ref. (35)); also see Fig. S2 for a darker exposure of GFP fluorescence scan [b] to more clearly visualize the difference in lane 1 *versus* lanes 2 and 3. We also captured a step preceding the pu-base, the Rpn14–Rpt6–Rpt3–Nas6 module, which was the fastest-migrating complex on a native gel (Fig. 3*B*, [b], t6-t3 module; Table S2). In both *rpt5-Δ3* and *nas2Δ* cells, a decrease in the pu-base was accompanied by the corresponding increase in the preceding Rpt6–Rpt3 module (Fig. 3*B*, [b], lanes 2 and 3), suggesting that Nas2 is needed for stable incorporation of this module into the pu-base.

Both the Rpt6–Rpt3 module and the pu-base complex were detected at a low level, supporting a view that these complexes may progress rapidly through base assembly and into the proteasome holoenzyme (17, 36). Also, both these complexes were resolved better *via* a 5% native gel as used in our experiments, than the more commonly used 3.5% for separating larger proteasomal complexes. These two features explain why these complexes may have not been readily detectable in previous studies.

### The pu-base forms during the ordered assembly of the base *via* chaperones

We examined how this poorly characterized and penultimate step of base assembly might be influenced by the actions of the other chaperones, which have been proposed to act together with Nas2 for proper assembly of the base complex (Fig. 3*A*) (12, 13, 17, 18, 30). For this, we left Nas2 intact, since it is required for the pu-base complex (Fig. 3*B*, lane 3) and deleted the other chaperones, *NAS6*, *RPN14*, and *HSM3*, individually or in combination. We then followed the Rpt6–Rpt3 module and its progression into the pu-base, by conducting GFP-3xFLAG-Rpt6 affinity purification and native gel analysis.

Whenever the Rpn14 chaperone in the Rpt6–Rpt3 module (Fig. 3*A*) was deleted alone, or together with another chaperone, this module was no longer detectable with little to no pu-base (Fig. 3*C*, [b, c], lanes 2, 3, 5, and 6). This result suggests that Rpn14 facilitates formation of the Rpt6–Rpt3 module and its progression into the pu-base, explaining a previously unknown feature of Rpn14 during early stage base assembly (34). By comparison, Nas6, the other chaperone in the Rpt6–Rpt3 module (Fig. 3*A*), seemed to be more important for the pu-base than the module itself. In the *nas6Δ* single mutants, the pu-base was not readily detectable although the Rpt6–Rpt3 module still existed (Fig. 3*C*, lane 8 in [b, c]). This result can be attributed to the known steric effect of Nas6, which may prevent a premature base complex, such as the pu-base, from incorporating into the proteasome holoenzyme (16, 19, 21, 22, 28). Without Nas6, the pu-base may prematurely proceed into the proteasome holoenzyme, depleting the cellular pool of pu-base. These results suggest that the penultimate step of base assembly relies on proper actions of not only Nas2 (Fig. 3*B*) but also Rpn14 and Nas6, which incorporate into this step from the preceding Rpt6–Rpt3 module during early stage base assembly.

To complete the base complex, the pu-base must associate with the incoming Rpt1–Rpt2–Rpn1 module *via* the Hsm3 chaperone (Fig. 3*A*). Without Hsm3, the Rpt1–Rpt2–Rpn1 module did not form efficiently (Fig. 3*E*, lanes 2 and 3 in [b]) (12, 14, 16, 17). This phenomenon explains some accumulation of both the pu-base and Rpt6–Rpt3 module in *hsm3Δ* cells (Fig. 3*C*, [b, c], lane 7), since these complexes cannot properly progress into the base, without the incoming Rpt1–Rpt2–Rpn1 module. When *HSM3* is deleted together with *RPN14* or *NAS6*, which are needed for the pu-base itself, little to no pu-base was detectable (Fig. 3*C*, [b, c], lanes 2, 4, and 5).

Taken together, our data demonstrate that the penultimate step of base assembly *via* Nas2 (Fig. 3*B*) is integrated with the actions of the other chaperones (Rpn14, Nas6, and Hsm3) (Fig. 3*C*). The steric effect of Nas2 may restrict the progression of the pu-base to ensure its proper subunit composition together with Rpn14 and Nas6, until the incorporation of the last module by Hsm3.

### Nas2 activates an assembly checkpoint, when Rpt1 cannot be properly incorporated *via* Hsm3

Next, we tracked Nas2 itself to examine how Nas2 may regulate base assembly with the other chaperones. We found Nas2 not only in the pu-base but also in previously unknown slow-migrating complexes, prominently in *hsm3Δrpn14Δ* cells and *hsm3Δ* cells (Fig. 3*C*, [a], see *arrowheads* in lanes 5 and 7). This result suggests that these additional Nas2-containing complexes may form, specifically upon recognizing defects in an Hsm3-mediated step during base assembly. Hsm3 is responsible for adding the final Rpt1–Rpt2–Rpn1 module to the pu-base, triggering Nas2 release for completion of the base (Fig. 3*A*). Thus, when the Hsm3-mediated step does not occur properly, Nas2 may not release, blocking further progression of the pu-base into the final step of base assembly. We will refer to this complex as Nas2–Check henceforth. Nas2–Check complexes may form at a low level, migrating at a similar position as RP as a more diffuse signal (Fig. 3*C*, lanes 5 and 7 in [a]).

We considered a scenario that the Nas2–Check complex might be an off-pathway or aggregated complex that forms simply because of the absence of Hsm3. However, this scenario is not supported by our data. First, the Nas2–Check complex requires the steric effect of Nas2, since this complex was no longer detected upon deletion of *NAS2*, as seen in *nas2Δ hsm3Δ* cells (Fig. S3, compare lane 3 to 2). Second and furthermore, Nas2 relies on another chaperone, Nas6, to maintain the Nas2–Check complex. This complex cannot form without Nas6, even when Nas2 is intact, as seen in *nas6Δhsm3Δ* and *nas6Δrpn14Δhsm3Δ* cells (Fig. 3*C*, [a], lanes 4 and 2). Nas6 may shield such a premature base complex by obstructing its association with the incoming CP until base assembly is complete (16, 19, 20). In support of this idea, when *NAS6* was deleted together with at least one or more chaperones, the cellular pool of base was noticeably depleted (Fig. 3*C*, [b], lanes 2, 3, and 4; see base). This result clarifies that simply the absence of Hsm3 does not result in the formation of the Nas2–Check complex. Rather, the Nas2–Check complex is specifically regulated *via* the combined steric effects of both Nas2 and Nas6, monitoring Hsm3 activity during base assembly, to ensure proper completion of the base before it incorporates into the proteasome.

If the Nas2–Check complex forms as a potential checkpoint upon loss of Hsm3 activity, this complex should not form upon compensating for the loss of Hsm3 activity. Since Hsm3 helps recruit Rpt1 into the final step of base assembly (Fig. 3*A*), we tested whether increasing Rpt1 expression might be sufficient to promote this final step, even without Hsm3 (16). For this, we introduced a low-copy Rpt1 expression plasmid into wildtype and *hsm3Δ* cells (Fig. 3*D*). In wildtype cells, there was no discernable effect on base assembly upon increased Rpt1 expression (Fig. 3*D*, [a, b], lanes 1 and 2). Notably, in *hsm3Δ* cells, the Nas2–Check complex was abolished upon increased Rpt1 expression (Fig. 3*D*, [a], compare lane 3 to 4). Also, the level of the base complex in *hsm3Δ* cells became more comparable to that in wildtype (Fig. 3*D*, [b], compare lane 4 to 1). Thus, if Rpt1 can efficiently incorporate at the final step of base assembly, Nas2 may release normally, allowing for completion of base assembly. These results explain the basis for the Nas2-dependent checkpoint, which Nas2 may distinguish normal *versus* defective base assembly, depending on whether Rpt1 can incorporate properly into the pu-base complex.

Next, we tested whether the Nas2-dependent checkpoint prevents incorporation of Rpt1, likely *via* steric hindrance, to block completion of the base complex (25, 26, 27). For this, we tested whether the Nas2–Check complex could be detected in the same complex with Rpt1 during base assembly. We conducted affinity purification using Rpt1 harboring GFP-3xFLAG tag upon deletion of *HSM3* and *NAS2* individually and together. In wildtype cells, Rpt1 incorporated into the Rpt1–Rpt2–Rpn1 module and then into the base, RP, and finally into the proteasome holoenzyme, as detected by Rpt1 GFP fluorescence (Fig. 3*E*, [b], lane 1). Upon deletion of *HSM3*, free Rpt1 became prominent, with the corresponding decrease in the Rpt1–Rpt2–Rpn1 module (Fig. 3*E*, [b], lanes 2 and 3) (12, 14, 16, 17). Importantly, the Nas2–Check complex was not detectable in any of Rpt1-containing complexes isolated from *hsm3Δ* cells, although it was readily detectable as a part of Rpt6-containing complexes in *hsm3Δ* cells (Fig. 3*E*, [a], compare lane 3 to 5). We in addition verified the absence of Nas2 in the Rpt1-containing complexes (Fig. S4, [c], lanes 1–4). These results demonstrate that the Nas2–Check complex cannot incorporate Rpt1, supporting our conclusion that the Nas2-dependent checkpoint prevents incorporation of Rpt1, likely *via* steric action, to block formation of a defective base complex.

### The Nas2-dependent checkpoint arises from the penultimate step of base assembly

As a readout for checkpoint activity during base assembly, the Nas2–Check complex migrates more slowly on native gels than the pu-base, which is normally the last step harboring Nas2 during base assembly (Fig. 3, *C*–*E*). We sought to examine whether the Nas2–Check complex arises from the pu-base. For this, we specifically tracked Nas2-containing complexes, using Nas2 itself as a bait for affinity purification in our panel of chaperone deletion strains.

**Figure 4:**
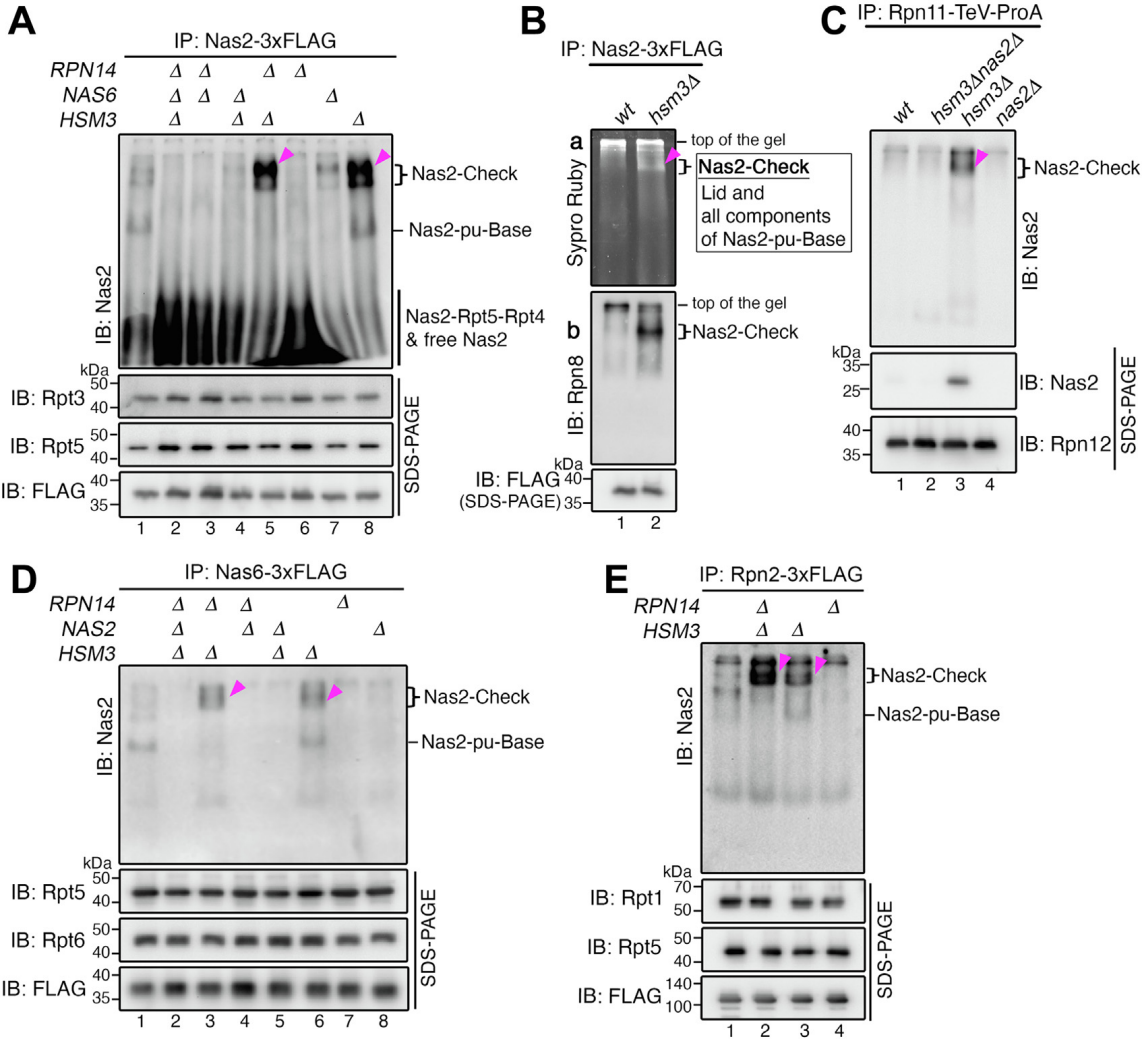
**The Nas2–checkpoint arises from the penultimate step of base assembly.***A*, Nas2–Check complexes (*arrowheads*) arise from the penultimate step of base assembly. Affinity purification was conducted, using 3xFLAG-tagged Nas2 as a bait. To detect the Nas2–Check complexes, Nas2 pull-down proteins (5 μg) were subjected to 5% native-PAGE and immunoblotting for Nas2. To confirm that largely comparable amounts of the complexes were analyzed, pull-down proteins (1 μg) were also subjected to 10% Bis–Tris SDS-PAGE followed by immunoblotting for two representative Rpt subunits. Each specific 3xFLAG-tagged bait protein for pulldown was also analyzed in the same manner, in panels *A*, *B*, *D*, and *E*. *B*, identification of the subunit composition of the Nas2–Check complex. The Nas2–Check complex was isolated from *hsm3Δ* cells using Nas2-3xFLAG as a bait and was resolved using native-PAGE as in *A*. The native gel was stained with Sypro Ruby [a] to excise the corresponding band for the Nas2–Check complex for proteomics analysis (Table S3). To validate the existence of the lid as a part of the Nas2–Check complex, the native gel as in [a] was subjected to immunoblotting for a representative lid subunit, Rpn8 [b]. Experiments were conducted as described in *A*. *C*, validation that the Nas2–Check complex is detected through reciprocal pulldown of the lid-containing complex. The lid-containing complexes were affinity purified using a representative lid subunit, Rpn11, harboring a TeV-ProA affinity tag. Further optimization was needed to detect the Nas2–Check complex in this pulldown (see the Experimental procedures section). To validate the presence of Nas2 in the lid-containing complexes, the isolated complexes were resolved using both native-PAGE and SDS-PAGE, as in *A*. To ensure that comparable amounts of the lid-containing complexes were analyzed, pull-down proteins (1 μg) were subjected to 10% Bis–Tris SDS-PAGE followed by immunoblotting for a lid subunit (Rpn12). The presence of Rpn12 also indicates the fully formed lid, since Rpn12 is the last subunit to incorporate into the lid (53). *D* and *E*, validation that the Nas2–Check complexes (*arrowheads*) arise from the pu-base, using two distinct components: the Nas6 chaperone and a non-ATPase subunit, Rpn2. Nas2–Check complexes are readily detectable in the pulldowns with Nas6-3xFLAG (*D*) as well as Rpn2-3xFLAG (*E*), upon analyzing 2.5 and 7.5 μg of the pull-down proteins, respectively. Experiments were conducted as described in *A*. Comparable amounts of the pull-down complexes were examined, as seen from two representative Rpt subunits and each specific 3xFLAG-tagged bait protein, upon analysis of the pull-down proteins (1 μg) using 10% Bis–Tris SDS-PAGE followed by immunoblotting.

Except the pu-base, Nas2–Check complexes were the only additional higher-order complex as evident from the native gel, and they were detected prominently in *rpn14Δhsm3Δ* and *hsm3Δ* cells (Fig. 4*A*, lanes 5 and 8; *arrowheads*). These results are consistent with those in our experiments using Rpt6 as a bait for tracking the Nas2–Check complex during base assembly (Fig. 3*C*, lanes 5 and 7; *arrowheads*). These data together support that the Nas2–Check complex arises directly from the penultimate step of base assembly. We noticed that the Nas2–Check complexes were detected as doublets (Fig. 4*A*, lanes 5 and 8, see an additional low-intensity signal below the arrowhead), suggesting that some small fraction of the Nas2–Check complexes might potentially exist in an additional form. Since the Nas2–Check complexes depend on the steric effect of Nas6 (Fig. 3*C*, [a], lanes 2 and 4), they were nearly undetectable in *nas6Δrpn14Δhsm3Δ* and *nas6Δhsm3Δ* cells (Fig. 4*A*, lanes 2 and 4); in these samples, Nas2 existed mostly in a Nas2–Rpt5–Rpt4 module and free Nas2. On the other hand, when Nas2–Check complex formed as in *rpn14Δhsm3Δ* and *hsm3Δ* cells, the Nas2–Rpt5–Rpt4 module and free Nas2 were decreased comparatively (Fig. 4*A*, lanes 5 and 8). Since the Nas2 chaperone is normally recycled by releasing from the completed base complex, disruption of its release because of Nas2–Check complex formation would interfere with such recycling to a new round of Nas2-dependent steps during base assembly.

Next, we determined the subunit composition of the Nas2–Check complex, by excising this band from a native gel, which was stained with Sypro Ruby (Fig. 4*B*, [a], lane 2). Based on our proteomics analysis, the Nas2–Check complex consisted of the fully formed lid and the pu-base (Table S3). This result can be explained by all interactions that normally occur between the lid and base subunits, as supported by several lines of evidence. First, the lid binds mainly three Rpt subunits (Rpt4, Rpt3, and Rpt6) through making extensive contacts with them (10). This feature explains how the lid can incorporate into the Nas2–Check complex, which lacks Rpt1 and Rpt2 (Tables S1 and S3). Second, a non-ATPase subunit, Rpn2, interacts with multiple lid subunits (10), explaining that Rpn2 in the Nas2–Check complex can also contribute to lid binding. Third, one function of the lid, independently of its deubiquitination activity, has been shown to stabilize the base complex in certain conformational states (37). Thus, the binding of the lid may stabilize the pu-base complex, until Rpt1 and Rpt2 can incorporate properly within the Nas2–Check complex.

To validate our proteomics data, we examined whether the lid complex could be detected as a part of the Nas2–Check complex. For this, we first immunoblotted our native gel with the Nas2–Check complex in it, using a representative lid subunit, Rpn8. The lid was indeed detected at the same position as the Nas2–Check complex (Fig. 4*B*, [b], lane 2). Reciprocally, when we isolated the lid-containing complexes, using Rpn11, a representative subunit of the lid, the Nas2–Check complex was detected, specifically in *hsm3Δ* cells (Fig. 4*C*, lane 3). Such a complex was no longer detectable upon *NAS2* deletion, as in *hsm3Δnas2Δ* cells, and was also not detectable in wildtype and *nas2Δ* cells (Fig. 4*C*).

Since our data suggest that the Nas2-dependent checkpoint utilizes the steric activity of Nas6 (Figs. 3*C* and 4*A*, lanes 2 and 4), we tested whether the Nas2–Check complex contained Nas6. Indeed, the Nas2–Check complex was readily detected in the Nas6 pulldown, specifically in *rpn14Δhsm3Δ* and *hsm3Δ* cells (Fig. 4*D*, lanes 3 and 6, see *arrowheads*). This result supports that the Nas2–Check complex contains Nas6, and the steric effect of Nas6 helps maintain this Nas2-dependent checkpoint. When *NAS2* was in addition deleted in these cells, the Nas2–Check complex was no longer detectable (Fig. 4*D*, lanes 2 and 5), confirming that the Nas2–Check complex also depends on the steric effect of Nas2 itself. To further verify that the Nas2–Check complex arises from the pu-base, we also conducted affinity purification using Rpn2 as a bait, since Rpn2, a non-ATPase subunit, is recruited into the pu-base later than the ATPase subunits, such as Rpt6, based on the proposed order of base assembly (Fig. 3*A*) (26, 27). Indeed, the Nas2–Check complex was detected in both *rpn14Δhsm3Δ* and *hsm3Δ* cells (Fig. 4*E*, lanes 2 and 3, see *arrowheads*). These results validate our proteomics data that all subunits in the pu-base complex exist in the Nas2–Check complex. Taken together, these data suggest that the Nas2-dependent checkpoint arises from the penultimate step of base assembly.

### Nas2 may recognize proper completion of base assembly *via* ATP hydrolysis by Rpt4

Our data explain how Nas2 sterically hinders Rpt1 addition at the checkpoint (Figs. 1, 2*C*, and 3, *C*–*E*) (25, 26, 27) and prompt us to examine how this same steric effect may also trigger Nas2 release, allowing Rpt1 addition to complete the base complex. This aspect is important for understanding how this checkpoint can be relieved *via* Nas2 release, suggesting that an additional feature might regulate the steric effect between Nas2 and Rpt1 during base assembly. We hypothesized that such a feature might be the fundamental activity of the assembled base—ATP hydrolysis, since it is initiated upon completion of the heterohexameric Rpt ring in the base (18) and is known to cause conformational changes in its subunits (6, 7, 8, 9). To test this hypothesis, we fixed individual Rpt proteins in an ATP-bound state by using *rpt-EQ* mutants; glutamine substitution for the conserved Walker B glutamate blocks ATP hydrolysis by a given Rpt protein (22, 38). Since the *rpt1*-*EQ*, *rpt4-EQ*, and *rpt5-EQ* mutants are lethal in yeast (38), they were expressed using plasmids in the presence of their endogenous chromosomal *RPT* gene to maintain cell viability. We predicted that the Nas2–check complex should form if ATP hydrolysis by any specific ATPase is necessary for Nas2 release and Rpt1 incorporation.

**Figure 5:**
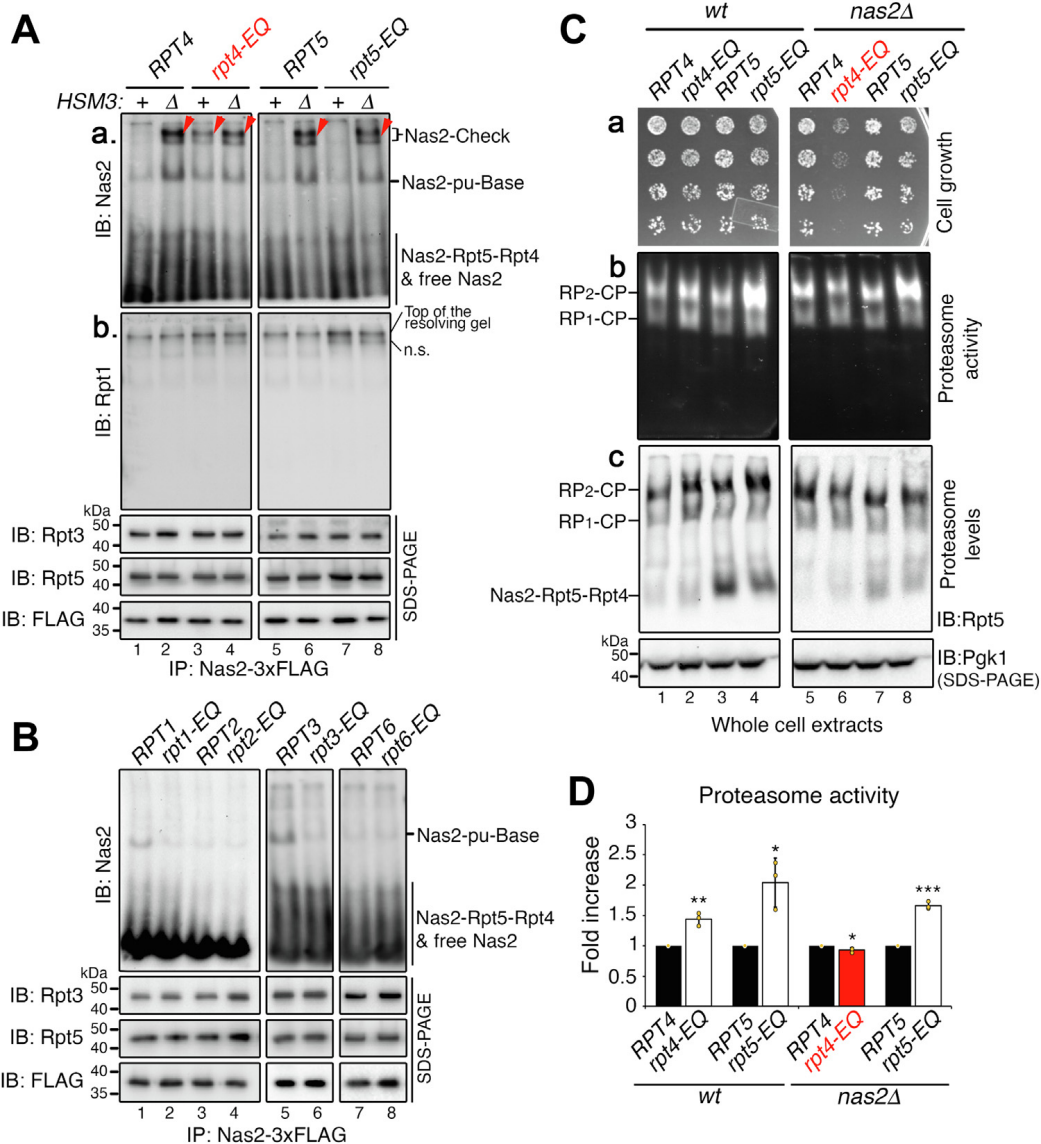
**Nas2 recognizes proper completion of base assembly *via* ATP hydrolysis by Rpt4.***A* and *B*, Nas2-dependent checkpoint is activated, specifically upon inhibiting ATP hydrolysis by Rpt4. Affinity purification was conducted using 3xFLAG-tagged Nas2. The purified proteins (5 μg) were subjected to 5% native-PAGE followed by immunoblotting for indicated proteins (ns, nonspecific signals; see text for details). To ensure that largely comparable amounts of the complexes were analyzed, pull-down proteins (4 μg) were also subjected to 10% Bis–Tris SDS-PAGE and immunoblotting for two representative Rpt subunits and the 3xFLAG-tagged Nas2 itself, as a bait for pulldowns. *C*, the Nas2-dependent checkpoint ensures efficient assembly of proteasome holoenzymes and cell growth upon heat stress. To assess heat sensitivity of the indicated yeast strains, threefold serial dilutions of yeast cells were spotted onto synthetic dropout media lacking leucine (a selection marker for the indicated plasmids) and were incubated at 37 °C for 2 to 3 days [a]. Proteasome activity and level were analyzed using yeast cultures upon heat stress at 37 °C for 3.5 h (see the Experimental procedures section for details). Whole-cell extracts (50 μg) were subjected to 3.5% native-PAGE and in-gel peptidase assays [b], followed by immunoblotting [c]. The Nas2–Rpt5–Rpt4 module is more noticeable in lanes 3 and 4 [c] since the total abundance of Rpt5 is increased because of combined plasmid-borne and chromosomal expression of *RPT5* allele, enhancing Rpt5 binding to its cognate Nas2 chaperone for the formation of this module. Pgk1, loading control. *D*, quantification of proteasome activity (RP_2_-CP) from data as in *C*, panel [b]. Fold increase of the total cellular proteasome activity in *rpt4-EQ* and *rpt5-EQ* (*C*, [b]) was calculated, relative to that of their wildtype counterpart (mean ± SD, n = 3 biological replicates, ∗*p* < 0.05, ∗∗*p* < 0.005, ∗∗∗*p* < 0.0005). See the Experimental procedures section for further details on quantification. CP, core particle; RP, regulatory particle.

Specifically, inhibition of ATP hydrolysis by Rpt4 resulted in the formation of Nas2–Check complexes (Fig. 5*A*, [a], lane 3; see *arrowhead*). Although Nas2 directly binds to Rpt5, a direct neighbor of Rpt4 during heterohexameric Rpt ring assembly (Fig. 3*A*), Nas2 released normally in the *rpt5-EQ* mutants (Fig. 5*A*, [a], lane 7). This result suggests that Nas2 release is not influenced by the nucleotide state of its cognate Rpt5, but by its direct neighbor Rpt4. In the *rpt5-EQ* background, the Nas2–Check complex formed only when *HSM3* was deleted as in *hsm3Δ rpt5-EQ* double mutants (Fig. 5*A*, [a], lane 8). Nas2 also released normally in *rpt1-EQ*, *rpt2-EQ*, *rpt3-EQ*, and *rpt6-EQ* mutants, as evidenced by no detectable Nas2–Check complex formation (Fig. 5*B*, lanes 2, 4, 6, and 8). These results suggest the specificity of ATP hydrolysis by Rpt4, as a requirement for Nas2 release for proper completion of the base complex.

The connection between Rpt4 ATP hydrolysis and Nas2 release may explain how Nas2 may recognize proper completion of base assembly. Nas2 specifically binds to Rpt5, which is positioned directly between Rpt4 and Rpt1, arranged as in Rpt4–Rpt5–Rpt1 in the heterohexameric Rpt ring of the base complex (Fig. 3*A*). As seen in *rpt4-EQ* cells, inhibiting Rpt4 ATP hydrolysis alone disrupted Nas2 release, although Hsm3 remained intact to recruit Rpt1 (Fig. 5*A*, [a], lane 3). This result suggests that Hsm3 activity alone may not be sufficient for ensuring both proper Rpt1 incorporation and Nas2 release, but in addition requires Rpt4 ATP hydrolysis for these events. If Rpt4 ATP hydrolysis is required only for Nas2 release, but not for Rpt1 addition, Rpt1 should be present in Nas2–Check complex specifically in *rpt4-EQ* cells. However, immunoblotting for Rpt1 exhibited only nonspecific signals, which were detected comparably in both *rpt4-EQ* and *rpt5-EQ* cells irrespective of the presence or the absence of Nas2–Check (Fig. 5*A*, [b], lane 3 *versus* 7, see ns); note that Nas2–Check did not form in the *rpt5-EQ* cells with intact Hsm3 (Fig. 5*A*, [a], lane 7). Thus, Rpt1 is not a component of Nas2–Check in *rpt4-EQ* cells, consistent with our data that Nas2–Check is not found in Rpt1-containing complexes (Fig. 3*E*, [a, c], lane 3).

Taken together, during heterohexameric Rpt ring assembly of the base, Nas2 may recognize Rpt4 ATP hydrolysis on one side of Rpt5 and Rpt1 incorporation to the other side of Rpt5. Upon proper completion of these two events on both sides of Rpt5, Nas2 may release, thereby ensuring correct completion of the base.

### The Nas2-dependent checkpoint ensures proper assembly of the proteasome holoenzyme

We examined to what extent the Nas2-dependent checkpoint might influence proteasome holoenzyme assembly in stressed conditions, such as heat, in which misfolded proteins are generated in a large quantity and require proteasome-mediated degradation for cell survival. When Nas2 is intact, both *rpt4-EQ* and *rpt5-EQ* cells tolerated heat stress at 37 °C, similarly to their corresponding control (Fig. 5*C*, [a], compare lanes 2 to 1 and 4 to 3). Both *rpt4-EQ* and *rpt5-EQ* cells increased their proteasome activities by 1.5-fold to 2-fold *via* increasing proteasome levels (Fig. 5*C*, [b, c], compare lanes 2 to 1 and 4 to 3; see Fig. 5*D* for quantification), because of a compensatory mechanism that raises proteasome gene expression upon deficit in proteasome function, such as defects in ATP hydrolysis as in these mutants (39, 40, 41).

Upon disabling the Nas2-dependent checkpoint by deletion of *NAS2*, the *rpt4-EQ* cells could not tolerate the heat stress, unlike the *rpt5-EQ* cells (Fig. 5*C*, [a], lane 6 *versus* 8). This growth defect of *rpt4-EQ nas2Δ* double mutant cells was due to a failure to increase proteasome holoenzyme assembly, since both the activity and level of the proteasome holoenzymes remained similar between the *rpt4-EQ nas2Δ* double mutants and their corresponding control (Fig. 5*C*, [b, c], compare lanes 6 to 5; see quantification in Fig. 5*D*). On the other hand, the *rpt5-EQ nas2Δ* double mutants exhibited an increase in proteasome activity by 1.7-fold, through increasing proteasome assembly, similarly to the extent seen between the *rpt5-EQ* single mutant and its corresponding control (Fig. 5*C*, [b, c], compare lanes 8 to 7 and lanes 4 to 3; see quantification in Fig. 5*D*). These results support that the Nas2-dependent checkpoint is specifically connected with Rpt4 ATP hydrolysis during base assembly, providing a mechanism crucial for proper and efficient formation of the proteasome holoenzyme.

## Discussion

### Nas2 monitors dual requirements for completion of base assembly

In the present study, we describe chaperone-mediated regulation at one of the least characterized steps of proteasome assembly, the completion of the base complex from the pu-base. This step needs to ensure whether the 9-subunit base formed properly, prior to its further progression into proteasome holoenzyme formation. To investigate this aspect of chaperone-mediated base assembly, we examined the Nas2 chaperone, based on the known correlation between Nas2 release and completion of the base complex (25, 26, 27).

Our findings reveal dual criteria by which Nas2 recognizes proper completion of the base complex: (1) addition of the last module, Rpt1–Rpt2–Rpn1 *via* the Hsm3 chaperone and (2) ATP hydrolysis by Rpt4 (Figure 3, Figure 4, Figure 5). Through these two tightly connected criteria, Nas2 may ensure not only correct subunit composition but also the functional competence of a newly assembled base. Such ability of Nas2 can be explained by its positioning, in that Nas2 directly binds to its cognate Rpt5, which exists between Rpt1 and Rpt4, as in Rpt1–Rpt5–Rpt4 in a heterohexameric Rpt ring (Fig. 3*A*). Thus, the Nas2 chaperone on Rpt5 can recognize whether Rpt1 is added properly to one side of Rpt5, based on ATP hydrolysis by Rpt4 on the other side of Rpt5 (Figs. 3 and 5). Since ATP hydrolysis is initiated upon completion of the heterohexameric Rpt ring (18), only the proper incorporation of Rpt1 may activate ATP hydrolysis by Rpt4, perhaps as the first event of ATP hydrolysis in a newly assembled base. Conformational changes by Rpt4 ATP hydrolysis may then transmit to the directly neighboring Rpt5, as a signal to release Nas2. In this way, Nas2 can release, only upon correct completion of the base both compositionally and functionally. Because our experimental system cannot yet distinguish between a true role of ATP hydrolysis *versus* a conformational impact, there is an alternative possibility that Nas2 release may be influenced by the conformational state of Rpt4 (or an effect on its neighbor, Rpt5), rather than ATP hydrolysis by Rpt4 directly. For example, the permanent ATP-bound state of Rpt4 in the *rpt4-EQ* mutant might induce an empty state in Rpt5, influencing Nas2 release, since the proteasomal ATPase ring has been proposed to undergo counterclockwise rotary hydrolysis (42, 43).

Since it is well established that ATP hydrolysis by the base causes specific conformational changes (6, 7, 8, 9), Rpt4 ATP hydrolysis may ensure proper base assembly not only by triggering Nas2 release but also by contributing to conformational changes essential for protein degradation. This idea is supported by the evidence that the *rpt4-EQ* base exhibits nonproductive conformational changes, abolishing the ability to degrade protein substrates by the proteasome holoenzyme, as seen from the recombinant base from the *E. coli* heterologous system (37). The *rpt4-EQ* mutation is lethal in yeast, further supporting the requirement of Rpt4 ATP hydrolysis for proteasome function (38). For example, the Rpt1–Rpt2–Rpn1 module, whose incorporation into the base is connected with ATP hydrolysis by Rpt4 (Fig. 5*A*), consists of subunits crucial for ubiquitin recognition and processing during protein degradation by the proteasome holoenzyme. Rpn1 is the major ubiquitin receptor, which recognizes polyubiquitinated protein substrates for the proteasome holoenzyme (44). Also, Rpn1 and Rpt1 together regulate the rate of protein degradation by controlling the activity of its bound Ubp6, a deubiquitinase of the proteasome (45, 46). These activities of Rpt1 and Rpn1 are tightly coordinated *via* conformational changes by the base complex, supporting the importance of Nas2 action in ensuring that the newly assembled base can undergo proper conformational changes.

### The Nas2-dependent checkpoint contributes to sequential checkpoints for base assembly

Our findings suggest that the Nas2-dependent checkpoint is activated, when one of the dual criteria for completion of base assembly is not satisfied, as seen in *hsm3Δ* and *rpt4-EQ* cells (Figure 3, Figure 4, Figure 5). At this checkpoint, Nas2 remains on the pu-base, instead of releasing from it and allowing its progression into the base. For this, Nas2 relies on another chaperone, Nas6, so that their combined steric effects against Rpt1 and CP, respectively, can maintain the Nas2–Check complex until proper assembly of the base, before incorporating into proteasome holoenzyme (Fig. 3*C* [a], lanes 2 and 4 and Fig. 4*D*, lanes 2 and 5). Although it is well agreed that Nas6 sterically hinders CP binding to ensure proper assembly of the base, this model was derived from studies mainly using the fully formed base (16, 19, 20). Our present study provides evidence supporting that Nas6 restricts the ongoing base assembly, as a part of the pu-base and the Nas2–Check complex, acting together with Nas2. In the Nas2–Check complex, only four Rpt subunits exist, meaning that they cannot yet form a stable ring-shaped complex. The lid may help stabilize such Nas2–Check complexes, while they can properly incorporate the remaining two Rpt subunits to complete the heterohexameric Rpt ring. This view is supported by the feature of the lid that it can stabilize the base in certain conformations (37). The lid might help ensure that Nas2–Check complex remains in an assembly-competent state for proper formation of the proteasome holoenzyme.

In many cancer cells, a human ortholog of Hsm3, S5b, is found to be silenced (47), exhibiting a situation as in *hsm3Δ* cells in our experiments. S5b silencing is suggested to be responsible for altered proteasome activity in these cancer cells (47). A future study will be needed to determine whether the Nas2-dependent checkpoint can be properly activated in cancer cells, and how it might influence proteasome assembly and functions, since this Nas2-dependent checkpoint is needed to satisfy an increased demand for proteasome holoenzyme assembly (Fig. 5, *C* and *D*).

An initial model of base assembly highlights that chaperones’ binding to ATPases sterically hinders the association of the base with the CP, until base assembly is complete (15, 16, 20, 23, 25). This model raises an important question: how might chaperones recognize proper completion and progression of the base into the proteasome? Combined with previous findings (21, 22, 28), our present study can help address this question. Nas2 can specifically recognize the nucleotide state of Rpt4 as a requirement for proper completion of the base (Fig. 5, *A* and *B*). As the base further matures into the proteasome holoenzyme, the other three chaperones (Rpn14, Hsm3, and Nas6) can individually distinguish the nucleotide state of specific Rpt subunits (21, 22, 28). These findings together propose a common feature of these chaperones that each chaperone may recognize specific criteria for proper completion of a given assembly step, based on the nucleotide state of specific Rpt subunits. Steric effects of each chaperone may be regulated through this mechanism, to distinguish normal *versus* defective assembly events. In this way, chaperones may provide a series of checkpoints, throughout the multistep assembly process of the base and its incorporation into the proteasome holoenzyme.

## Experimental procedures

### Yeast strains, plasmids, and biochemical reagents

A complete list of yeast strains and plasmids are provided in Tables S4 and S5, respectively. Unless specified otherwise, all yeast strains were cultured in yeast extract–peptone–dextrose medium at 30 °C. Yeast strains harboring specific gene expression plasmids were grown in synthetic dropout medium lacking the appropriate auxotrophic marker for the plasmids at 30 °C. All yeast manipulations were conducted using standard procedures (48). Yeast strains harboring multiple engineered alleles were generated through crossing two appropriate strains, followed by sporulation and dissection. When the same markers were used for two alleles, PCR was used to distinguish strains with each of those two alleles.

At least two biological replicates were performed for all biochemical and imaging experiments. Details of biochemical reagents are included in each specific Experimental procedures section. A complete list of antibodies is provided in Table S6. All antibodies were used at 1:3000 dilutions except anti-Pgk1, which was used at 1:10,000 dilution.

### Affinity purification of the base and pu-base from the heterologous *E. coli* system

*E. coli* BL21-star (DE3) cells were cotransformed with three plasmids, which were obtained from Andreas Martin’s laboratory (29): pCOLA-1 (FLAG-Rpt1, Rpt2, His_6_-Rpt3, Rpt4, Rpt5, and Rpt6), pETDuet-1 (Rpn1, Rpn2, and Rpn13), and pACYCDuet-1 (Nas2, Nas6, Hsm3, and Rpn14). Genes for rare tRNAs were also included in the pACYCDuet-1 plasmid (29). In Figure 2*A* (lane 2), Nas2 in the pACYCDuet-1 plasmid has a premature stop codon at amino acid 65. In Figure 2*A* (lane 4) and Figure 2*C* (lanes 2 and 4), the pCOLA-1 plasmid contains Rpt5 with the last five amino acids deleted. All plasmids are listed in Table S5. *E. coli* cells were grown in 35 ml LB media at 37 °C overnight and were inoculated into 2 l fresh LB media next day at an absorbance of 0.05 at 600 nm and were grown to an absorbance of 0.6 at 600 nm. Cultures were cooled to room temperature and were induced using 1 mM IPTG at 18 °C in water bath overnight. Cells were harvested by centrifugation at 4000*g* for 10 min at 4 °C. Cell pellets were washed once with one pellet volume of cold lysis buffer (50 mM sodium phosphate [pH 7.0], 300 mM NaCl, and 10% glycerol), which is supplemented with 1 mM β-mercaptoethanol, and were centrifuged at 4000*g* for 10 min. Cell pellets were resuspended using the residual buffer. The resulting cell resuspension was frozen in a drop-wise manner into liquid nitrogen and was ground using a mortar and pestle in the presence of liquid nitrogen. The ground cryopowders were hydrated with three volumes of lysis buffer containing 1 mM β-mercaptoethanol, protease inhibitors, and ATP (1 mM), on ice for approximately 20 min with intermittent vortexing. ATP (1 mM) was included for the rest of the purification procedure. Typically, 2 l cultures generate 15 ml cryopowders, which are then hydrated with 45 ml of lysis buffer. Triton X-100 was added to 0.2% final to aid the solubilization of the proteins on ice for 10 min. Cleared lysates were obtained by centrifugation at 20,000*g* at 4 °C for 30 min.

To isolate the pu-base using His_6_-Rpt3 as a bait, the cleared lysates were incubated with 250 μl of TALON metal affinity resin (Clontech; catalog no.: 635502) for 1 h at 4 °C. Beads-bound proteins were collected by centrifugation at 3000 rpm for 5 min, followed by three washes with five beads volume of lysis buffer containing 0.2% Triton X-100, using filter column. The pu-base was eluted from the beads using three beads volume of lysis buffer containing 150 mM imidazole by rotating for 1 h at 4 °C.

To isolate the base complex using FLAG-Rpt1 as a bait, the cleared lysates were incubated with 250 μl of anti-FLAG M2-agarose beads (Sigma; catalog no.: A2220-5ML) for 2 h at 4 °C. The beads were washed three times with five beads volume of proteasome buffer containing 150 mM NaCl. The base complex was eluted using three beads volume of proteasome buffer containing 0.2 mg/ml FLAG peptides (GLP Bio; catalog no.: GP10149-5) for 1 h at 4 °C. The eluates were concentrated using a 30,000 molecular weight cutoff concentrator (Amicon; catalog no.: UFC503024).

### Affinity purification of endogenous proteasomal and chaperone-bound complexes

Overnight yeast cultures were inoculated at an absorbance of 0.25 at 600 nm in 400 ml yeast extract–peptone–dextrose. Cultures were grown an absorbance of 2 at 600 nm and were harvested by centrifugation at 3000*g* for 5 min. Cell pellets were washed once with cold water, frozen in liquid nitrogen, and ground with mortar and pestle in the presence of liquid nitrogen. The ground cryopowders were hydrated for 10 min on ice using proteasome buffer (50 mM Tris–HCl [pH 7.5], 5 mM MgCl_2_, 150 mM NaCl, 1 mM EDTA, 10% glycerol, and protease inhibitors). ATP was added to 1 mM in all buffers used throughout the experiments. Hydrated lysates were centrifuged at 20,000*g* for 30 min at 4 °C. Affinity purification using a 3xFLAG affinity tag to each specific bait was conducted by incubating the cleared lysates with 30 μl of anti-FLAG M2-agarose beads (Sigma; catalog no.: A2220-5ML) for 2 h at 4 °C. The beads were washed twice with 400 μl of proteasome buffer containing 150 mM NaCl with 1 mM ATP and eluted in three bead volumes of proteasome buffer containing 0.2 mg/ml FLAG peptides (GLP Bio; catalog no.: GP10149-5) for 1 h at 4 °C.

In Figure 4*B* for proteomics analysis, the Nas2–Check complex was obtained *via* FLAG affinity purification as described previously, except that different buffers were used as follows. The ground cryopowders were hydrated using Hepes buffer (60 mM Hepes [pH 7.6], 50 mM NaCl, 50 mM KCl, 5 mM MgCl_2_, 0.5 mM EDTA, and 10% glycerol) containing 0.2% NP-40. Bead-bound materials were washed twice with the Hepes buffer containing 0.1% NP-40. For elution with FLAG peptides, Hepes buffer without NP-40 was used. These Hepes buffers were also used in Figure 4*C*, where affinity purification using a TeV-ProA tag to Rpn11 was conducted by incubating the cleared lysates with 30 μl IgG resin (MP Biomedicals; catalog no.: 855961) for 2 h at 4 °C. The beads were washed twice using the Hepes buffer containing 0.1% NP-40. Elution was conducted in three bead volumes of Hepes buffer containing 1 μl of tobacco etch virus protease (Promega; catalog no.: PRV6101) for 1 h at 37 °C. Proteomics analysis (Table S3) was conducted specifically for the band corresponding to Nas2–Check (Fig. 4*B*, [a], lane 2, *arrowhead*); a control gel slice was not included since any discrete band was not readily discernible in our control lane on the Sypro Ruby-stained gel (Fig. 4*B*, [a], lane 1).

### Native-PAGE analysis

Affinity-purified chaperone-bound complexes (the amount specified in each figure legend) were loaded onto 5% discontinuous native gels containing a 2.5% stacking portion. Native gels were electrophoresed for 4.5 h at 100 V in the cold room. To visualize proteasomal complexes containing GFP-tagged Rpt6 or Rpt1, native gels were imaged using Amersham Typhoon scanner (GE Healthcare Bio-Sciences AB), using Cy2 filter at a pixel size of 100 μm.

For experiments in Figure 5*C*, whole-cell lysates (50 μg) were loaded onto 3.5% continuous native gels and electrophoresed for 3 h at 100 V in the cold room. In-gel peptidase assays were conducted using the fluorogenic peptide substrate LLVY-AMC (Bachem; catalog no.: I-1395.0100) as described previously (32). Native gels were photographed under UV light Bio-Rad Gel Doc EZ Imager to detect AMC fluorescence. For quantification in Figure 5*D*, these photographs as in Figure 5*C* [b] were analyzed using ImageJ software (NIH) (49) to measure the signal intensities of the LLVY-AMC hydrolytic activities by the RP_2_–CP complex, a major form of the proteasome holoenzyme complex in each specific lane. Fold increase was calculated for each *rpt4-EQ* and *rpt5-EQ* mutant, relative to their corresponding *RPT4* and *RPT5* control. The graph indicates the fold change in total cellular proteasome activity as depicted in Figure 5*C* [b], resulting from total cellular proteasome levels in Figure 5*C* [c]; our loading control, Pgk1, is equal in all samples. Proteasome activity is not normalized to proteasome level, since such a graph would be more appropriate for assessing whether *rpt4-EQ* or *rpt5-EQ* mutation directly affects peptidase activity of the proteasome. This aspect was largely ruled out by a previous study showing that *rpt4-EQ* and *rpt5-EQ* mutants have little to no effects on peptidase activity, since their peptidase stimulation rates are *rpt4-EQ* (95% wildtype) and *rpt5-EQ* (118% wildtype), based on real-time solution assays tracking LLVY-AMC hydrolysis (29).

### Heat stress of yeast cell cultures

Overnight yeast cultures were diluted to an absorbance of 0.25 at 600 nm in synthetic media lacking leucine (marker for plasmids used in Fig. 5*C*) and were grown to approximately absorbance of 1 at 600 nm at 30 °C. These cultures were diluted again to an absorbance of 0.3 at 600 nm in fresh synthetic media lacking leucine in a total volume of 100 ml and further incubated at 37 °C for 3.5 h to allow for at least two doublings, as in previous studies (30, 34, 36). Cells were harvested as described in the “Affinity purification of endogenous proteasomal and chaperone-bound complexes” section, except that whole-cell extracts were obtained by centrifuging the hydrated cryolysates at 15,000*g* twice for 15 min each in the cold room.

### Immunoblotting

Polyvinylidene difluoride membranes (MilliporeSigma, Immobilon; catalog no.: IPVH00010) were used to transfer proteins from SDS-PAGE or native-PAGE gels. Blocking buffer was prepared by adding 5% nonfat dry milk to TBST (Tris-buffered saline with 0.1% Tween-20). About 20 ml of blocking buffer was used to incubate with the polyvinylidene difluoride membrane for 1 h at room temperature. Two washes were conducted for 10 min each, using TBST. Primary antibodies were diluted in blocking buffer at 1:3000 dilution (except Pgk1 at 1:10,000 dilution) and incubated with SDS-PAGE membranes for 1 h at room temperature. For native-PAGE membranes, this step was conducted overnight at 4 °C for all primary antibodies, except for anti-Rpt5 antibody (1 h, room temperature). Two washes were conducted using TBST as aforementioned. Antimouse immunoglobulin G horseradish peroxidase–linked antibody (Cytiva; catalog no.: NA931) or anti-rabbit immunoglobulin G horseradish peroxidase–linked antibody (Cytiva; catalog no.: NA934) was diluted at 1:3000 in blocking buffer and incubated with the membranes for 1 h at room temperature, followed by two washes using TBST. Membranes were then subjected to Enhanced chemiluminescence (PerkinElmer; Western Blot Chemiluminescence Reagents Plus, NEL105001EA) and were imaged using Bio-Rad ChemiDoc MP Imager.

## Data availability

All data are contained within the article.

## Supporting information

This article contains supporting information (50, 51, 52).

## Conflict of interest

The authors declare that they have no conflicts of interest with the contents of this article.
