# Supplementary material for: The penultimate step of proteasomal ATPase assembly is mediated by a switch dependent on the chaperone Nas2
Source: J Biol Chem. 2023 Jan 5;299(2):102870. doi: 10.1016/j.jbc.2023.102870 (PMC9922823; doi:10.1016/j.jbc.2023.102870)
Supplement: Supporting Figures S1–S4 and Tables S1–S6 [file mmc1.pdf]

Supporting Information

The Penultimate Step of Proteasomal ATPase Assembly is Mediated by a Switch Dependent on the Chaperone Nas2

Suganya Sekaran and Soyeon Park

Supporting Information contains 4 figures and 6 tables.

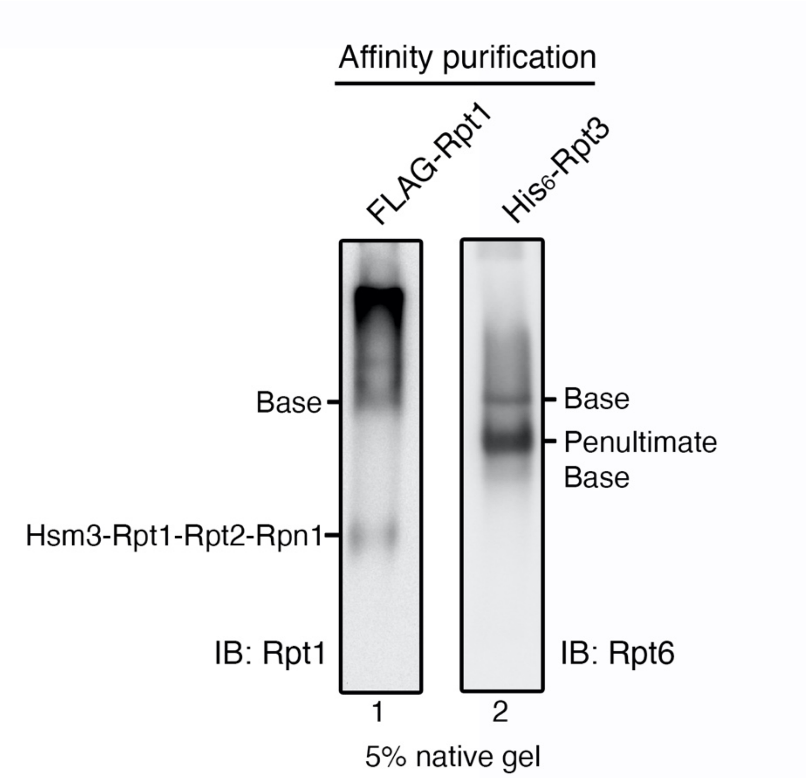

Figure S1. Additional characterization of the FLAG-Rpt1 and His$_6$-Rpt3 affinity-purification from the heterologous *E. coli* system.

Affinity-purification was conducted using FLAG-Rpt1 or His$_6$-Rpt3 as a bait, as described in Fig. 1. Upon native gel analysis of FLAG-Rpt1 affinity-purified complexes, the base complex was detected (lane 1; also see Fig. 1, lane 1), together with an additional Rpt1-containing complex, which is likely to be the preceding Hsm3-Rpt1-Rpt2-Rpn1 module, as seen from immunoblotting for its subunit, Rpt1 (lane 1). Upon native gel analysis of His$_6$-Rpt3 affinity-purified complexes, the penultimate base complex was detected (lane 2; also see Fig. 1, lane 2), but the preceding Rpn14-Rpt6-Rpt3-Nas6 module was not detected when examined by immunoblotting for its component, Rpt6, likely due to its rapid progression into the penultimate base complex.

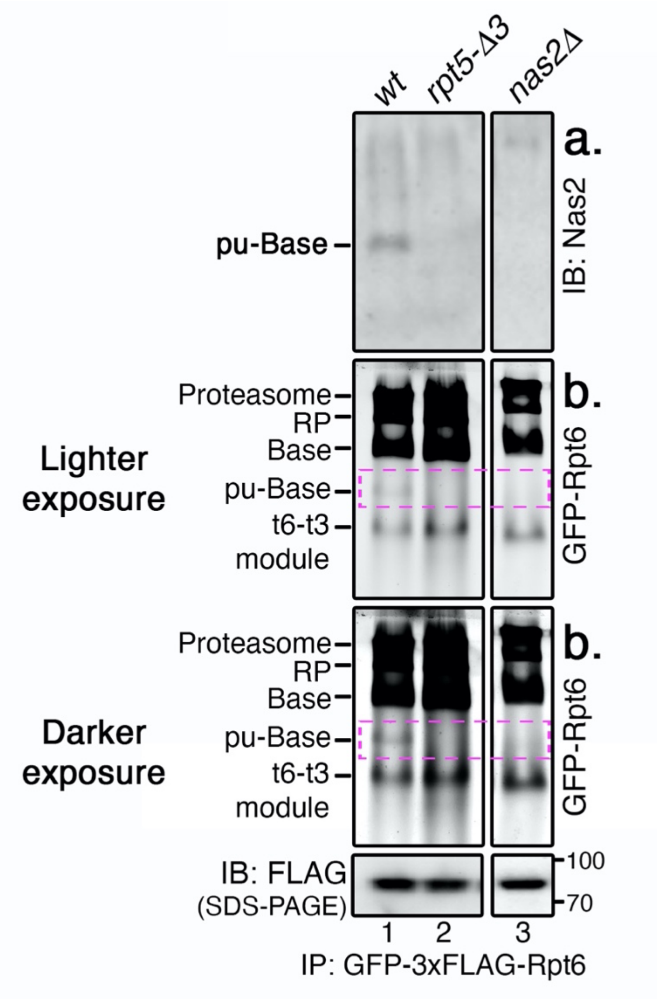

Figure S2. Main text Figure 3B is shown with additional data to more clearly visualize that the existence of the penultimate base depends on Nas2 binding to Rpt5 or Nas2 itself.

Main text Figure 3B is shown, together with a darker exposure of the panel **[b]**, GFP fluorescence scan. The darker exposure helps more clearly visualize that the penultimate base is not detected upon disruption of Nas2 binding to Rpt5 (*rpt5-Δ3*) or deletion of Nas2 itself (*nas2Δ*), by its absence from the dotted pink box. In main text Figure 3B, the lighter exposure of panel **[b]** is shown, to visualize the penultimate base, together with more abundant late-stage assembly intermediates (base and RP) as discrete bands. Lane 3 (*nas2Δ*) is identical to lane 4 from Figure S3, and is shown to provide a side-by-side comparison for lane 2 (*rpt5-Δ3*), to confirm that the penultimate step of base assembly relies on Nas2 binding to Rpt5 (*rpt5-Δ3*) or Nas2 itself (*nas2Δ*).

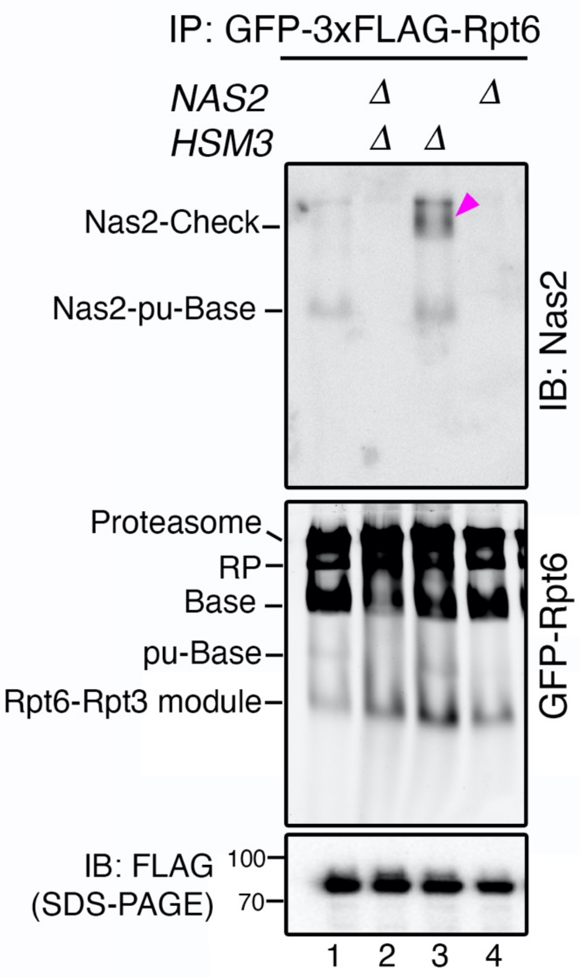

Figure S3. Validation that the Nas2-Check complex requires the Nas2 chaperone.

The Nas2-Check complex in the *hsm3Δ* cells (lane 3, arrowhead) forms in a Nas2-dependent manner, as it is not detected in the *nas2Δhsm3Δ* double mutants (lane 2). Affinity-purification was conducted using a 3xFLAG affinity tag appended to Rpt6. The purified proteins (7.5 μg) were then subjected to 5% native-PAGE and immunoblotting for Nas2, and GFP fluorescence detection to visualize all Rpt6-containing complexes. Anti-FLAG immunoblot, loading control for FLAG affinity purification.

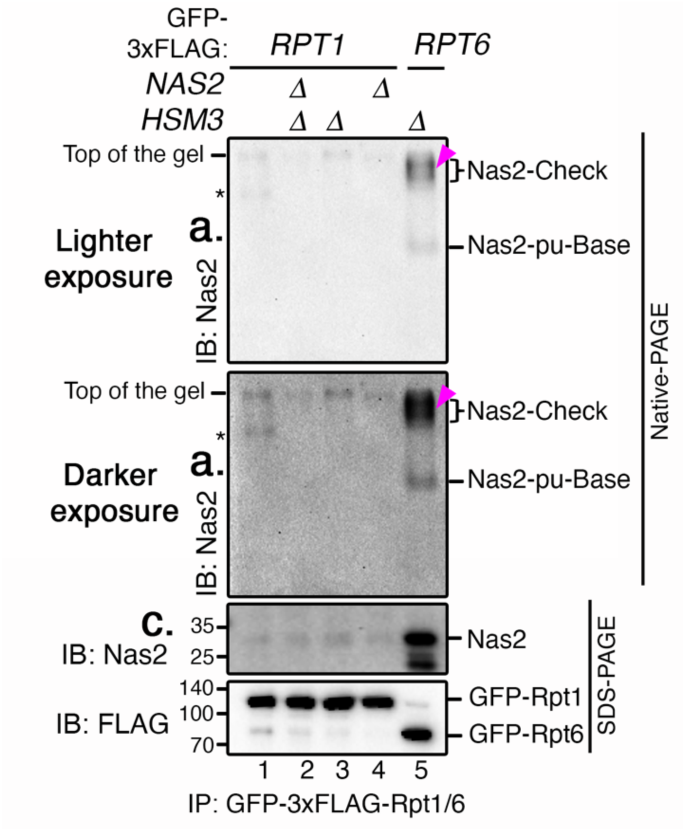

Figure S4. Main text Figure 3E is shown with additional data, supporting that Rpt1 cannot incorporate into the Nas2-Check complex, due to steric hindrance of Nas2 against Rpt1.

Main text Figure 3E is shown, with 1) a darker exposure of the panel **[a]** and 2) an immunoblot for Nas2 in **[c]**, to clarify that the Nas2-Check complex does not harbor Rpt1 (lanes 1-4 in **[a, c]**), consistent with our proteomics analysis of the Nas2-Check complex (Table S3). In **[a]**, some faint bands are detected at the top of the resolving gel and at the asterisk. Both are detected at positions different from the Nas2-Check (arrowhead), suggesting that they are not Nas2-Check complexes. Furthermore, Nas2 is not detected in these samples (lanes 1 through 4 in **[c]**) upon immunoblotting of the SDS-PAGE membrane, except some shadows seen in these lanes, including *nas2Δ* samples. Nas2 is detected only in our positive control (lane 5, [**a** and **c**]), where the Nas2-Check complex is isolated via Rpt6, a component of this complex.

Table S1. Base and penultimate base isolated from *E. coli* heterologous system in Fig. 1.

		Subunit Name	No. of unique peptide	No. of total peptide
Base complex	Base subunits	*RPN1*	47	336
		RPN2	56	388
		RPN13	6	74
		RPT1	30	382
		RPT2	29	175
		RPT3	22	223
		RPT4	28	203
		RPT5	30	115
		RPT6	25	262
	Chaperones	*NAS6*	11	59
		RPN14	20	194
		HSM3	24	183
Penultimate base complex	Base subunits	*RPN2*	23	26
		RPN13	4	7
		RPT3	30	348
		RPT4	34	285
		RPT5	33	273
		RPT6	27	418
	Chaperones	*NAS6*	15	87
		RPN14	21	364
		NAS2	13	109

Table S2. Endogenous penultimate base and Rpt6-Rpt3 module in Fig. 3B

		Subunit Name	No. of unique peptide	No. of total peptide
Endogenous penultimate base complex	Base subunits	*RPN2*	5	5
		RPT3	4	5
		RPT4	6	6
		RPT5	8	8
		RPT6	9	9
	Chaperones	*NAS6*	3	3
		RPN14	8	9
		NAS2	4	4
Endogenous Rpt6-Rpt3 module	Base subunits	*RPT3*	6	9
		RPT6	7	7
	Chaperones	*NAS6*	3	3
		RPN14	7	8

Table S3. Nas2-Check complex in Fig. 4B

	Subunit Name	No. of unique peptide	No. of total peptide
Lid subunits	*RPN3*	13	13
	RPN5	17	17
	RPN6	13	13
	RPN7	15	16
	RPN8	12	12
	RPN9	20	20
	RPN11	9	9
	RPN12	5	5
Base subunits	*RPN2*	21	25
	RPT3	10	11
	RPT4	14	14
	RPT5	19	19
	RPT6	11	11
Chaperones	*NAS6*	5	5
	RPN14	6	6
	NAS2	5	5

Table S4. Yeast strains used in this study

Strain	Genotype	Source
SUB62[a]	*MATa lys2-801 leu2-3, 2-112 ura3-52 his3-Δ200 trp1-1*	(50)
SP2017	*MATa rpt6::Prpt6-yEGFP1F-RPT6-LEU2*	(34)
SP4210A	*MATa rpt6::Prpt6-yEGFP1F-RPT6-LEU2. rpt5::rpt5-Δ3 (kanMX6)*	This study
SP3027A	*MATα rpt6::Prpt6-yEGFP1F-RPT6-LEU2. nas2::kanMX6*	This study
SP4316A	*MATa rpt6::Prpt6-yEGFP1F-RPT6-LEU2 rpn14::hphMX4. hsm3::kanMX6 nas6::HIS3*	This study
SP2963A	*MATα rpt6::Prpt6-yEGFP1F-RPT6-LEU2 rpn14::hphMX4. nas6::HIS3*	(34)
SP4317	*MATa rpt6::Prpt6-yEGFP1F-RPT6-LEU2 hsm3::kanMX6 nas6::HIS3*	This study
SP4268A	*MATa rpt6::Prpt6-yEGFP1F-RPT6-LEU2 rpn14::hphMX4. hsm3::kanMX6*	This study
SP2964B	*MATα rpt6::Prpt6-yEGFP1F-RPT6-LEU2 rpn14::hphMX4*	(34)
SP3026A	*MATa rpt6::Prpt6-yEGFP1F-RPT6-LEU2 hsm3::KAN*	(34)
SP2965C	*MATa rpt6::Prpt6-yEGFP1F-RPT6-LEU2 nas6::his3*	(34)
SP3025A	*MATa rpt6::Prpt6-yEGFP1F-RPT6-LEU2 nas2::KAN hsm3::KAN*	(34)
SP2781A	*MATa rpt1::Prpt1-yEGFP1F-RPT1-LEU2*	(34)
SP3033A	*MATa rpt1::Prpt1-yEGFP1F-RPT1-LEU2 nas2::KAN*	(34)
SP3032A	*MATa rpt1::Prpt1-yEGFP1F-RPT1-LEU2 hsm3::KAN*	(34)
SP3031A	*MATa rpt1::Prpt1-yEGFP1F-RPT1-LEU2 nas2::KAN hsm3::KAN*	(34)
SP1655A	*MATa nas2::NAS2-6×Gly-3×FLAG (kanMX6)*	(21)
SP3210	*MATα nas2::NAS2-6×Gly-3×FLAG(kanMX6) nas6::HIS3 rpn14::hphMX hsm3::KAN*	This study
SP3198A	*MATa nas2::NAS2-6×Gly-3×FLAG (kanMX6) nas6::HIS3 rpn14::hphMX*	This study
SP3196	*MATa nas2::NAS2-6×Gly-3×FLAG (kanMX6) nas6::HIS3 hsm3::KAN*	This study
SP3194	*MATα nas2::NAS2-6×Gly-3×FLAG (kanMX6) rpn14::hphMX hsm3::KAN*	This study
SP3200A	*MATa nas2::NAS2-6×Gly-3×FLAG (kanMX6) rpn14::hphMX*	This study
SP3202	*MATα nas2::NAS2-6×Gly-3×FLAG (kanMX6) nas6::HIS3*	This study
SP3193	*MATα nas2::NAS2-6×Gly-3×FLAG (kanMX6) hsm3::KAN*	This study
SP1677A	*MATa nas6::NAS6-6×Gly-3×FLAG (hphMX)*	(21)
SP3128A	*MATα nas6::NAS6-6×Gly-3×FLAG (hphMX) rpn14::hphMX hsm3::KAN nas2::NAT*	(30)
SP2692A	*MATa nas6::NAS6-6×Gly-3×FLAG (hphMX) rpn14::hphMX hsm3::KAN*	(30)
SP3124A	*MATa nas6::NAS6-6×Gly-3×FLAG (hphMX) rpn14::hphMX nas2::NAT*	(30)
SP3127A	*MATa nas6::NAS6-6×Gly-3×FLAG (hphMX) hsm3::KAN nas2::NAT*	(30)
SP3129A	*MATα nas6::NAS6-6×Gly-3×FLAG (hphMX) hsm3::KAN*	(30)
SP1883A	*MATa nas6::NAS6-6×Gly-3×FLAG (hphMX) rpn14::hphMX*	(30)
SP3132A	*MATα nas6::NAS6-6×Gly-3×FLAG (hphMX) nas2::NAT*	(30)
SP1493A	*MATa rpn2::RPN2-3×FLAG:HIS3*	This study
SP4458A	*MATa rpn2::RPN2-3×FLAG:HIS3 rpn14::hphMX hsm3::KAN*	This study
SP4457A	*MATa rpn2::RPN2-3×FLAG:HIS3. hsm3::KAN*	This study
SP4456B	*MATa rpn2::RPN2-3×FLAG:HIS3. rpn14::hphMX*	This study
sDL133	*MATa rpn11::RPN11-TEV-ProA (HIS3)*	(51)
SP3111A	*MATα rpn11::RPN11-TEV-ProA (HIS3) hsm3::KAN nas2::NAT*	This study
SP3109A	*MATa rpn11::RPN11-TEV-ProA (HIS3) hsm3::KAN*	This study
SP3107A	*MATa rpn11::RPN11-TEV-ProA (HIS3) nas2::NAT*	This study
SP4245A	*MATa nas2::NAS2-6×Gly-3×FLAG (kanMX6) rpt6::HIS3. [YCplac33-RPT6]*	This study
SP4246A	*MATa nas2::NAS2-6×Gly-3×FLAG (kanMX6) rpt3::HIS3. [YCplac33-RPT3]*	This study
SP1654B	*MATa nas2::KAN*	(30)

[a]All strains are isogenic to SUB62 genetic background.

Table S5. Plasmids used in this study

Name	Description	Source and Reference
pAM82	FLAG-Rpt1, Rpt2, His_6-Rpt3, Rpt4, Rpt5, Rpt6 in pCOLA-1	Martin Laboratory (29)
pAM83	Rpn14, Nas6, Nas2, Hsm3 and tRNAs for rare codons in pACYCDuet-1	Martin Laboratory (29)
pAM81	Rpn1, Rpn2, Rpn13 in pETDuet-1	Martin Laboratory (29)
N/A	FLAG-Rpt1, Rpt2, His_6-Rpt3, Rpt4, Rpt5 without last 5 amino acids, Rpt6 in pCOLA-1	Martin Laboratory (29)
pSP310	Rpn14, Nas6, Nas2 with a premature stop codon, Hsm3 and tRNAs for rare codons in pACYCDuet-1	This study
pSP151	His_6-Rpt5 in pRSF-Duet-1	(30)
pSP158	Untagged Rpt4 in pRSF-Duet-1	(30)
pSP128	GST-Nas2 in pGEX6P-1	(30)
pJR751	pRS316-*RPT1*	Roelofs Laboratory (34)
pRT357	pRS314-*RPT1*	Tomko Laboratory (38)
pRT702	YCplac111-*RPT2*	Tomko Laboratory (26)
pRT364	YCplac111-*RPT3*	Tomko Laboratory (26)
pRT1528	YCplac111-*RPT4*	Tomko Laboratory (38)
pRT1408	pRS315-*RPT5*	Tomko Laboratory (38)
pRT1496	YCplac111-*RPT6*	Tomko Laboratory (38)
pRT1409	pRS314-*rpt1(E310Q)*	Tomko Laboratory (38)
pRT1410	YCplac111-*rpt2(E283Q)*	Tomko Laboratory (38)
pRT1411	YCplac111-*rpt3(E273Q)*	Tomko Laboratory (38)
pRT1529	YCplac111-*rpt4(E283Q)*	Tomko Laboratory (38)
pRT1413	pRS315-*rpt5(E282Q)*	Tomko Laboratory (38)
pRT1497	YCplac111-*rpt6(E249Q)*	Tomko Laboratory (38)

Table S6. Antibodies used in this study

Name	Source and reference
Rabbit polyclonal Anti-Nas2	Roelofs Laboratory (31)
Rabbit polyclonal Anti-Rpt5	Enzo Life Sciences, BML-PW8245
Rabbit polyclonal Anti-Rpt3	Enzo Life Sciences, BML-PW8250
Rabbit polyclonal Anti-Rpt6	Carl Mann laboratory (52)
Rabbit polyclonal Anti-Rpt1	Carl Mann laboratory (52)
Rabbit polyclonal Anti-Rpn14	Finley laboratory (16)
Rabbit polyclonal Anti-Rpn8	Finley laboratory (16)
Rabbit polyclonal Anti-Rpn12	Finley laboratory (16)
Mouse monoclonal Anti-FLAG	Sigma, F3165
Mouse monoclonal Anti-His	Sigma, H1029
Mouse monoclonal Anti-Pgk1	Life Technologies, 459250

References

50. Finley, D., Ozkaynak, E., and Varshavsky, A. (1987) The yeast polyubiquitin gene is essential for resistance to high temperatures, starvation, and other stresses. *Cell* **48**, 1035-1046

51. Leggett, D. S., Hanna, J., Borodovsky, A., Crosas, B., Schmidt, M., Baker, R. T., Walz, T., Ploegh, H., and Finley, D. (2002) Multiple associated proteins regulate proteasome structure and function. *Mol Cell* **10**, 495-507

52. Ghislain, M., Udvardy, A., and Mann, C. (1993) S. cerevisiae 26S protease mutants arrest cell division in G2/metaphase. *Nature* **366**, 358-362
